# miR-196B-5P and miR-200B-3P Are Differentially Expressed in Medulloblastomas of Adults and Children

**DOI:** 10.3390/diagnostics10050265

**Published:** 2020-04-29

**Authors:** Michela Visani, Gianluca Marucci, Dario de Biase, Felice Giangaspero, Francesca Romana Buttarelli, Alba Ariela Brandes, Enrico Franceschi, Giorgia Acquaviva, Alessia Ciarrocchi, Kerry Jane Rhoden, Giovanni Tallini, Annalisa Pession

**Affiliations:** 1 Department of Specialized, Diagnostic and Experimental Medicine, Anatomic Pathology-Molecular Diagnostic Unit AUSL-IRCCS of Bologna, University of Bologna School of Medicine, 40138 Bologna, Italy; giorgia.acquaviva3@unibo.it (G.A.); giovanni.tallini@unibo.it (G.T.); 2 Anatomic Pathology Unit, Ospedale Bellaria AUSL-IRCCS of Bologna, 40139 Bologna, Italy; Gianluca.Marucci@istituto-besta.it; 3 Department of Pharmacy and Biotechnology (FaBiT), Molecular Diagnostic Unit AUSL of Bologna, University of Bologna, 40138 Bologna, Italy; annalisa.pession@unibo.it; 4 Department of Radiological, Oncological and Anatomo-Pathological Sciences, Sapienza University School of Medicine, 00161 Rome, Italy; felice.giangaspero@uniroma1.it; 5 IRCCS Neuromed, 86077 Pozzilli (Isernia), Italy; 6 Department of Human Neurosciences, Sapienza University School of Medicine, 00161 Rome, Italy; francesca.buttarelli@uniroma1.it; 7 Department of Medical Oncology, Bellaria–Maggiore Hospitals AUSL-IRCCS of Bologna, 40139 Bologna, Italy; a.brandes@ausl.bologna.it (A.A.B.); enricofra@yahoo.it (E.F.); 8 Laboratory of Translational Research, Arcispedale Santa Maria Nuova AUSL-IRCCS of Reggio Emilia, 42122 Reggio Emilia, Italy; Alessia.Ciarrocchi@ausl.re.it; 9 Department of Medical and Surgical Sciences, Medical Genetics Unit, University of Bologna School of Medicine, 40138 Bologna, Italy; kerry.rhoden@unibo.it

**Keywords:** microRNA, microRNA profile, adult medulloblastoma, childhood medulloblastoma

## Abstract

Medulloblastoma is a highly aggressive brain tumor that typically affects children, while in adults it represents ~1% of all brain tumors. Little is known about microRNA expression profile of the rare adult medulloblastoma. The main aim of this study was to identify peculiar differences in microRNA expression between childhood and adult medulloblastoma. Medulloblastomas were profiled for microRNA expression using the Exiqon Human miRNome panel (I + II) analyzing 752 microRNAs in a training set of six adult and six childhood cases. Then, the most differentially expressed microRNAs were validated in a total of 21 adult and 19 childhood cases. Eight microRNAs (miR-196b-5p, miR-183-5p, miR-200b-3p, miR-196a-5p, miR-193a-3p, miR-29c-3p, miR-33b-5p, and miR-200a-3p) were differentially expressed in medulloblastoma of adults and children. Analysis of the validation set confirmed that miR-196b-5p and miR-200b-3p were significantly overexpressed in medulloblastoma of adults as compared with those of children. We followed an in silico approach to investigate direct targets and the pathways involved for the two microRNAs (miR-196b and miR-200b) differently expressed between adult and childhood medulloblastoma. Adult and childhood medulloblastoma have different miRNA expression profiles. In particular, the differential dysregulation of miR-196b-5p and miR-200b-3p characterizes the miRNA profile of adult medulloblastoma and suggests potential targets for novel diagnostic, prognostic, or therapeutic strategies.

## 1. Introduction

Medulloblastoma (MB) represents about 10% of pediatric brain tumors in children from birth to 14 years old [1,2,3], with the highest incidence in children aged three to ten years [4]. In contrast, MB in adults (patients ≥ 16 years of age) represents about 1% of all adult brain tumors [4,5,6]. The difference in the incidence between adults and children suggests distinct origins and tumorigenic mechanisms. Indeed, many studies have confirmed that MBs in adults and children are different types of tumors regarding their histology, localization, genetic landscape, and risk stratification [7,8,9,10,11].

Presently, MBs are classified into the following four molecular subgroups with peculiar cytogenetic, mutational, and gene expression characteristics [12]: (i) medulloblastoma WNT-activated (characterized by enhanced WNT-beta catenin pathway activation); (ii) medulloblastoma SHH-activated and *TP53*-mutant (characterized by activation of the SHH pathway and mutation in *TP53* gene); (iii) medulloblastoma SHH-activated and *TP53*-wildtype (characterized by activation of the SHH pathway and absence of mutations in *TP53* gene); and (iv) medulloblastoma non-WNT/non-SHH (Group 3, Group 4). [13]. This classification was incorporated into the latest World Health Organization (WHO) Classification of Tumors of the Central Nervous System [12] and has become a widely accepted criterion for MB diagnosis and to direct specific therapeutic strategies [12,13].

Although the same classification is applied to MBs of children and adults, several studies have reported that the SHH molecular subtype was preponderate among adults, while the non-WNT/non-SHH (Group 3) seems to be primarily restricted to pediatric age groups [14,15].

MicroRNAs are small (18–24 nt) non-coding RNAs that negatively regulate the expression of several mRNA targets. It is well established that miRNAs have distinct expression profiles in different tissues and have essential roles in the physiologically regulation of cell functions. Deregulation of miRNAs expression has a crucial impact on the control of cell growth, contributing to the development of cancer [16,17].

Important correlations between miRNA profiles and MB molecular subgroups or histological subtypes have been described in the literature. Moreover, a potential predictive role has been proposed for some miRNAs [18,19,20,21,22,23,24,25,26,27,28,29,30,31,32,33,34,35,36,37,38,39,40,41,42,43,44].

The main aim of this study was to compare miRNA expression in childhood and adult MB and to find miRNAs that were differentially expressed between these two groups. We identified peculiar differences in miRNA expression between childhood and adult tumors, and we showed that miR-196b-5p and miR-200b-3p are significantly overexpressed in MB of adults.

## 2. Materials and Methods

### 2.1. Ethics Statement

This study was approved by the institutional review board of the Azienda USL of Bologna, Italy (CE: 09113; Prot. N. 1241/CE, 22 September 2010). All cases were retrieved and managed following the ethics committee’s guidelines (CE: 09113). All experiments were approved by the review board, and they were carried out following relevant guidelines and regulations (CE: 09113).

Our institutional review board (Azienda USL, Bologna, Italy) approved the study also in the absence of written informed consent because it was a retrospective study, and all samples were anonymized.

All information regarding human material was managed using anonymous numerical codes, and all samples were handled in compliance with the Helsinki Declaration (https://www.wma.net/policies-post/wma-declaration-of-helsinki-ethical-principles-for-medical-researchinvolving-human-subjects/).

### 2.2. Patient Samples

Formalin-fixed and paraffin-embedded (FFPE) MB samples were retrospectively retrieved from the archives of the Anatomic Pathology Unit of Bellaria Hospital (Bologna). MBs were histologically re-classified according to the 2016 WHO classification [12]: medulloblastoma WNT-activated (WHO code 9475/3 [12]), medulloblastoma SHH-activated and *TP53*-mutant (WHO code 9476/3 [12]), medulloblastoma SHH-activated and *TP53*-wildtype (WHO code 9471/3 [12]), and medulloblastoma non-WNT and non-SHH (WHO code 9477/3 [12]). The study involved 40 cases consisting of 26 males (65%) and 14 females (35%), aged from 1 to 66 years. Cases were divided into two groups according to age, i.e., adults (>16 years) and children (0–16 years). The adult cases (AD) comprised 21 tumors (mean age 30.48 years, range 16–66 years), childhood cases (CH) 19 tumors (mean age 6.58 years, range 1–15 years). Twelve cases (6 adults and 6 children) were selected as a training set, while all 40 cases were used as the validation set. Clinical information was obtained from existing medical records following institutional guidelines, and all data were handled with anonymous numerical codes.

### 2.3. Nucleic Acid Extraction

All histology sections were reviewed, and the most representative was selected, marking the areas of cancer with at least 90% of tumor content for molecular analysis. Four 20 μm sections and two 10 μm sections were used for RNA and DNA extraction, respectively. Tumor material marked from each section was manually dissected. DNA was extracted using a QuickExtract FFPE DNA year.Extraction Kit (Epicentre, Madison, WI, USA). Total RNA enriched with miRNA was extracted using a RecoverAll Total Nucleic Acid Isolation Kit for FFPE (Thermo Fisher Scientific, Waltham, MA, USA), according to the manufacturer’s instructions. Qubit DNA BR Assay Kit and Qubit RNA HS Assay Kit were used to evaluating DNA and RNA quantity (Thermo Fisher Scientific, Waltham, MA, USA), respectively.

### 2.4. Immunostaining

Immunohistochemistry (IHC) was carried out by the streptavidin-biotin-immunoperoxidase technique on 3 μm sections of the FFPE samples. Molecular subgroups of MB were identified by IHC using a combination of four antibodies, and different conditions were used for each one as follows: anti-β-catenin (clone beta-catenin-1, Agilent, Santa Clara, CA, USA) 1:150; anti-Filamin A (clone FLMN01, PM6/317, Thermo Fisher Scientific, Waltham, MA, USA) at 1:500, anti-GAB1 (clone EPR375, Ab133486, Abcam, Cambridge, UK) at 1:300, and anti-YAP1 (clone 63.7, sc101199, Santa Cruz Biotechnology, Inc., Dallas, TX, USA) at 1:200.

β-Catenin was scored as positive when ≥10% of neoplastic nuclei where stained, regardless of cytoplasmic immunoreactivity. GAB1, YAP1, and filamin A were scored as positive when ≥20% of neoplastic cells showed staining intensity >1. Granular nuclear reactivity was considered as negative. Samples were assigned to the WNT molecular subgroup when β-catenin nuclear translocation or mutation in *CTNNB1* exon 3 were present; GAB1, YAP1, and filamin A triple positivity identified the SHH molecular subgroup; negativity for all the previous biomarkers account for molecular subgroup non-WNT and non-SHH.

### 2.5. CTNNB1 and TP53 Mutational Screening

Exon 3 of *CTNNB1* of all cases, and exons 4, 5, 6, 7, 8, and 9 of *TP53* of SHH subgroup samples, were sequenced using a TruSeq Custom Amplicon panel run on a MiSeq Illumina instrument (Illumina Inc., San Diego, CA, USA), according to established protocols [45].

### 2.6. Mirnome Expression Analysis

Six AD cases and six CH cases were selected as the training sets, and miRNA expression patterns were evaluated using the Exiqon miRCURY LNA™ Human Panels (I + II) RT-PCR (Exiqon, Vedbæk, Denmark). Specimens were all normalized to the same concentration, and AD and CH cases were pooled into AD-RNA and CH-RNA pools, respectively. cDNA was synthesized using a universal cDNA Synthesis Kit II (Exiqon, Vedbæk, Denmark). Diluted cDNA was mixed with ExiLENT SYBR^®^ Green master mix (Exiqon, Vedbæk, Denmark). Quantitative real-time PCR (RT-qPCR) was performed using a Roche LightCycler^®^ 480 Real-Time PCR system (Roche, Basel, Switzerland). The analysis was performed in duplicate, according to the manufacturer’s instructions, and the differential miRNA expression between AD and CH groups was evaluated by the global mean normalization method.

### 2.7. MicroRNAs Validation

On the basis of the miRnome screening findings, a subset of 8 miRNAs (miR-196b-5p, miR-183-5p, miR-200b-3p, miR-196a-5p, miR-193a-3p, miR-29c-3p, miR-33b-5p, and miR-200a-3p) was selected for validation by RT-qPCR. In addition, miR-191-5p and miR-320a were included as reference genes. MicroRNA validation was performed for all selected cases (21 AD and 19 CH).

Total RNA from tumor samples was reverse transcribed using the TaqMan Advanced miRNA cDNA Synthesis Kit (Thermo Fisher Scientific, Waltham, MA, USA), according to the manufacturer’s instructions. Quantification of miRNAs was performed in triplicate of 10 μL reactions using predesigned TaqMan Advanced miRNA Assays (Thermo Fisher Scientific, Waltham, MA, USA) and TaqMan Fast Advanced Master Mix (Thermo Fisher Scientific, Waltham, MA, USA) on the Light Cycler instrument (Roche), according to the manufacturer’s instructions. The results were analyzed by the multiple reference normalization method, using miR-191-5p and miR-320a as reference genes.

### 2.8. miRNAs Target Prediction and Pathway Enrichment

miRTarBase (http://mirtarbase.mbc.nctu.edu.tw/php/index.php, “hsa MTI.xls” file, release 7.0 of 15th September 2017) and miRWALK (miRWALK 3.0, http://129.206.7.150/, updated November 2017) were used to search mRNA targets of selected miRNAs. We restricted our analysis only to experimentally validated targets. Targets were, then, analyzed and grouped according to pathway classification using the PANTHER web tool (Version 13.0 released 2017-11-12, http://www.pantherdb.org/) [46,47] and according to their molecular function and biological process involvement using g:Profiler (http://biit.cs.ut.ee/gprofiler/) [48].

A further enrichment analysis for pathway classification was performed using Enrichr web tool (https://amp.pharm.mssm.edu/Enrichr/, [49,50]), focusing the attention on “KEGG 2019 Human” pathway analysis.

### 2.9. Target Differentially Expressed in Adult vs. Childhood MBs

In silico prediction was performed using data available on the GEO datasets database (https://www.ncbi.nlm.nih.gov/geo/). Research was performed using the following MeSh terms: “medulloblastoma”, “expression profiling”, and “homo sapiens OR human”. Only series with the following criteria were used for the analysis: (i) tissue samples (no cell lines); (ii) availability of data about age of patients and diagnosis; and (iii) both childhood and adult MBs must be tested. According to these criteria, then, a total of 3 datasets were selected as follows: GSE41842 [43], GSE49243 [51,52], and GSE21140 [53] (Table 1 and Supplementary Table S1).

Each dataset was analyzed using the Geo2R tool, splitting the available specimens into two groups, i.e., children MBs (≤16 years) and adult MBs (>16 years).

The most significantly downregulated target (logFC ≤ −1 and *p* < 0.05) in adult MBs were then compared with genes targeted by miR-196b-5p and miR-200b-3p. Then, the genes common to both groups were analyzed using in silico pathway analysis using the Enrichr web tool.

### 2.10. Statistical Analysis

All analyses were performed using GenEx Professional (version 6.0, Multid Analyses AB) and GraphPad Prism (version 6.01, GraphPad Software, Inc., La Jolla, CA, USA) tools.

To minimize potential noise, miRNAs with a cycle threshold (*C*t) value greater than or equal to “39” were considered as undetected. Expression values and fold changes, relative to AD groups versus CH groups, were obtained by relative quantification and the 2^−ΔΔ*C*t^ method [54]. Unsupervised hierarchical clustering analysis was generated considering Euclidean correlation and average linkage. Gaussian distribution was evaluated by the Kolmogorov–Smirnov test. For the identification of differentially expressed miRNAs between different groups and subgroups, Student’s t-test or the Mann–Whitney test were used depending on the results of the Kolmogorov–Smirnov normality test. A microRNA was considered to be downregulated with a median fold change (FC) < −2.0, while a microRNA with a median FC ≥ 2.0 was deemed to be upregulated. The level of significance was *p* < 0.05 for all statistical analyses.

For the microRNA target analyses, a statistical overrepresentation test was performed using the PANTHER web tool (http://www.pantherdb.org/tools/uploadFiles.jsp). The list of target genes was compared to a reference list to determine statistically over- or underrepresentation of PANTHER classification categories. This binomial statistical test was applied to analyze the PANTHER pathway classifications and *p*-values were calculated with the Bonferroni correction; a cut-off of 0.05 was considered to estimate if a specific PANTHER category was over- or underrepresented in a statistically significant way.

An additional in silico pathway analysis was performed using the Enrichr web tool (https://amp.pharm.mssm.edu/Enrichr). The list of target genes was compared to a reference list to determine which pathways were principally involved. The combined score, that is, a combination of the *p*-value and z-score, was used for sorting enriched terms (https://amp.pharm.mssm.edu/Enrichr/, [49,50]).

*p*-values were determined with the Fisher’s exact test, and rank score or z-score were computed using a modification to the Fisher’s exact test in which a z-score for deviation from an expected rank was calculated. The combined score was computed by taking the log of the *p*-value from the Fisher exact test and multiplying it by the z-score of the deviation from the expected rank.

Gene ontology analysis, for molecular enrichment functions and biological processes, was performed using the g.Profiler web tool. In particular g:Cocoa (http://biit.cs.ut.ee/gprofiler/gcocoa.cgi) was used to rank and compare gene lists through their functional annotations. The analysis was performed excluding electronically inferred annotations from enrichment analysis (“no electronic GO annotations” was enabled). For the statistical analysis of GO annotation, the g:SCS algorithm was selected, developed by g:Profiler, since its simulations provided a better threshold between significant and nonsignificant results than FDR or Bonferroni Correction [48]. A *p*-value cut-off of 0.05 was considered.

### 2.11. Construction of Protein–Protein Interaction (PPI) Network

Unraveling the protein–protein interaction (PPI) of miRNA targets is essential to elucidate molecular mechanisms. The PPI network was evaluated by STRING v10.5 (http://string.-db.org) [55]. A confidence score >0.4, was set as the cut-off criterion. Each network node represents a specific miRNA target, and the edges represent the predicted functional associations. To investigate the functional associations, we selected the following active interaction sources: “text mining” (a function showing a list of significant protein interaction groups, extracted from the abstracts of scientific literature); “databases” (a feature showing a list of significant protein interaction groups, gathered from curated databases); and “experiment” (a function showing a list of significant protein interaction datasets, gathered from other protein–protein interaction databases).

The network was visualized using Cytoscape [56]. CytoHubba plug-in was used to calculate the degree (i.e., the number of interactions) of each node in the PPI network.

### 2.12. Data Availability

The data that support the findings of this study are available from the corresponding author upon reasonable request.

## 3. Results

### 3.1. Patients Subgrouping

According to a histological review, 21 of 40 tumors (52.5%) were classified as classical (CL) MB (nine in the AD group and 12 in the CH group), 13 cases (32.5%) were classified as desmoplastic/nodular (D/N) (nine in the AD group and four in the CH group), and six (15%) were classified as large cells anaplastic (LCA) MB (three in the AD group and three in the CH group) (Table 2).

**Figure 1:**
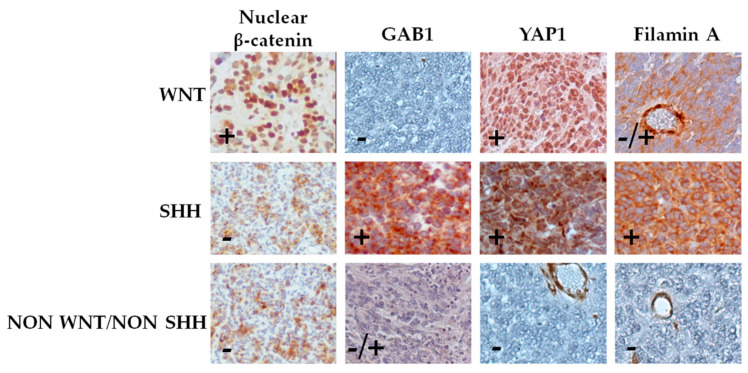
Classification of medulloblastoma by immunohistochemical staining. Cases were classified into WNT subgroup (nuclear β-catenin, positive; GAB1, negative; and YAP1, positive), SHH subgroup (nuclear β-catenin, negative; GAB1, positive; YAP1, positive; and filamin A, positive), and non-SHH/WNT subgroup (nuclear β-catenin, negative; YAP1, negative; and filamin A, negative). Representative pictures (40×) of each subgroup are shown.

According to immunohistochemistry (Figure 1) and *CTNNB1* mutational status (Supplementary Table S2), as indicated in the WHO guidelines [12], samples were divided into the following subgroups: WNT, SHH TP53-wildtype, and non-WNT/non-SHH (Table 2).

**Figure 2:**
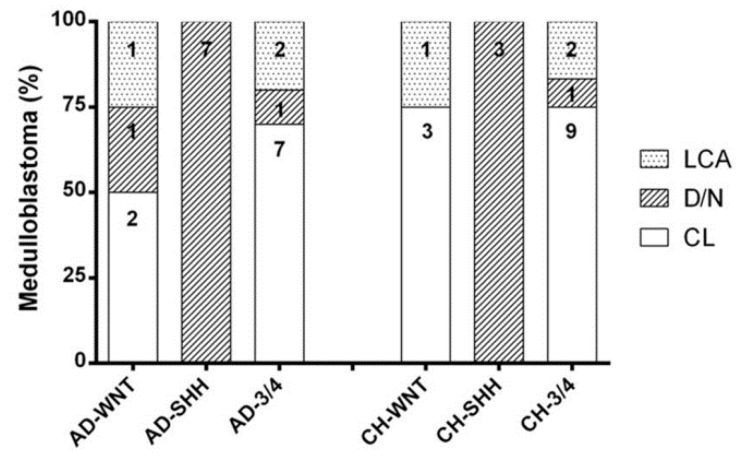
Histopathological distribution in adult and children molecular subgroups. Each column shows the number of cases in each category.

In the AD group, four samples belonged to the WNT subgroup (AD-WNT), seven to the SHH subgroup (AD-SHH), and ten samples to the non-WNT/non-SHH subgroup (AD non-WNT/non-SHH) (Table 2 and Figure 2).

Histologically, the AD-WNT subgroup was composed of two “CL” (50.00%), one “D/N” (25.00%), and one “LCA” MB (25.00%), all AD-SHH cases were of the “D/N” subtype; among AD non-WNT/non-SHH subgroup cases, seven were “CL” (70.00%), one “D/N” (10.00%), and two “LCA” (20.00%) MBs (Table 2 and Figure 2).

In the CH group, four samples belonged to the WNT subgroup (CH-WNT), three samples to the SHH subgroup (CH-SHH), and the remaining 12 samples to the non-WNT/non-SHH subgroup (CH non-WNT/non-SHH) (Table 2 and Figure 2).

Histologically, the CH-WNT subgroup was composed of three “CL” (75.00%) and one “LCA” (25.00%) subtypes; all CH-SHH cases were “D/N”; among the CH non-WNT/non-SHH subgroup cases, nine were “CL” (75.00%), one “D/N” (8.33%), and two “LCA” (16.67%) subtypes (Table 2 and Figure 2).

All SHH cases, both from the AD and CH groups, were *TP53* wild-type and were accordingly classified as SHH-activated/TP53-wildtype following the WHO 2016 [12].

Three out of four CH-WNT cases presented a non-canonical *CTNNB1* mutation in exon 3 codon 13 (p.A13V), codon 20 (p.A20T), and codon 81 (p.D81N). None of these mutations involved the hot spot region between codons 29 and 51, which encoded for the phosphorylation site of GSK-3β (Supplementary Table S2). Immunohistochemical evaluation of these *CTNNB1* mutated cases did not show nuclear protein accumulation. These cases were included in the CH-WNT subgroup anyway, due to the presence of *CTNNB1* mutation in exon 3.

### 3.2. miRNome Expression Analysis in the Adult and Childhood Medulloblastoma

A comprehensive screening of 752 miRNAs with Exiqon miRCURY PCR panels revealed distinct microRNA expression signatures in the training set that discriminated between AD- and CH-miRNA pools (Table 3).

Compared to the CH group, the most upregulated miRNAs in the AD group were miR-196b-5p, miR-200b-3p, miR-193a-3p, miR-376a-3p, and miR-451a, whereas the most downregulated miRNAs were miR-183-5p, miR-642a-5p, miR-182-5p, miR-196a-5p, and miR-96-5p (Table 3).

According to this training set, we selected eight miRNAs (miR-196b-5p, miR-196a-5p, miR-193a-3p, miR-183-5p, miR-200a-3p, miR-200b-3p, miR-29c, and miR-33b-5p). This list includes five that were among the top most differentially expressed miRNA shown in Table 3 (miR-196b-5p, miR-196a-5p, and miR-193a-3p overexpressed in the AD versus the CH group; miR-183-5p and miR-200b-3p underexpressed in AD versus CH group), and there were three that were expressed only in the AD group (miR-29c-3p, miR-33b-5p, and miR-200a-3p). No miRNAs were significantly expressed in the CH group but not in AD group.

### 3.3. Validation of the Subset of Eight miRNAS in the Adult and Childhood Medulloblastoma Groups

**Figure 3:**
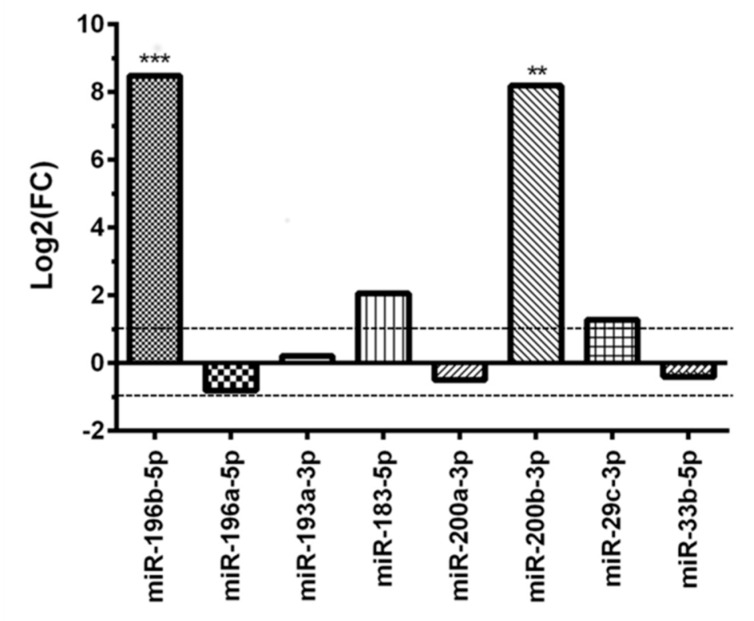
Differential miRNAs expression in the adult MB group versus the childhood MB group after validation test. ** *p* < 0.01 and *** *p* < 0.001.

The expression of the subset of eight miRNAs, identified in the training set, was measured in the validation set. As reported in Figure 3, only miR-196b-5p and miR-200b-3p were differentially expressed, both with significant overexpression in the AD versus the CH group (*p* < 0.001 and *p* < 0.01, respectively).

MiR-183-5p and miR-29c-3p showed a not statistically significant dysregulation in the AD group (*p* = 0.9352 and *p* = 0.6163, respectively), whereas miR-196a-5p resulted slightly downregulated (*p* = 0.3861) (Figure 3).

### 3.4. miRNAs Expression in Molecular Subgroups of Adult Medulloblastoma

**Figure 4:**
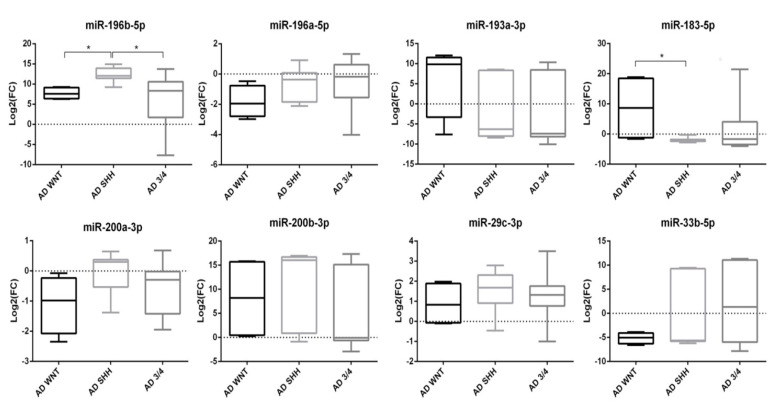
Boxplot of miRNAs expression in molecular subgroups of adult medulloblastoma. Log2(FC) was calculated on CH group. * *p* < 0.05.

To better define a miRNA signature for each adult MB molecular subgroup, cases were divided into WNT, SHH, and non-WNT/non-SHH subgroups. Compared to the whole CH group, AD-SHH was characterized by upregulation of miR-196b-5p (*p* < 0.001) and miR-200b-3p (*p* < 0.01) (Figure 4).

Moreover, upregulation of miR-196b-5p was still observed following normalization against CH-SHH cases (*p* < 0.05).

Mir-196b-5p (*p* < 0.05), miR-183-5p (*p* < 0.05), and miR-200b-3p (*p* < 0.05) resulted upregulated in the AD-WNT subgroup, as compared with the whole CH group (Figure 4). Normalized on the CH-WNT cases, in the AD-WNT group only, two miRNAs (miR-183-5p and miR-200b-3p) resulted upregulated with a level of deregulation which was near to statistical significance (*p* = 0.057 and *p* = 0.057, respectively). Moreover, one miRNA (miR-33b-5p) was downregulated but in a not-statistically significant way (*p* = 0.200).

In the AD non-WNT/non-SHH subgroup, only the upregulated miR-196b-5p reached the significance threshold (*p* < 0.05) as compared with the whole CH group (Figure 4). Comparing AD non-WNT/non-SHH with CH non-WNT/non-SHH cases, miR-196b-5p resulted significantly upregulated (*p* < 0.05).

Considering the molecular subgroups classification of adult MB (WNT, SHH, and non-WNT/non-SHH), comparing each group with each other, miR-196b-5p was overexpressed in the AD-SHH subgroup versus the AD-WNT (*p* < 0.05) and AD non-WNT/non-SHH subgroups (*p* < 0.05) (Figure 4). On the contrary, miR-183-5p was downregulated in the AD-SHH subgroup as compared with the AD-WNT (*p* < 0.05) (Figure 4).

Taking into consideration the histological subgroups of medulloblastoma, miR-196b-5p was overexpressed in AD-D/N MB as compared with its expression in the CH-D/N groups (*p* < 0.01).

Comparing the AD-CL and CH-CL subgroups, only miR-196b-5p, miR-183-5p, and miR-200b-3p resulted upregulated, but without reaching statistical significance (data not shown).

Five miRNAs (miR-196b-5p, miR-183-5p, miR-200b-3p, miR193a-3p, and miR-29c-3p) resulted upregulated even if in a not statistically significant way (data not shown) when AD-LCA MB and the CH-LCA subgroups were compared.

### 3.5. Analysis of the Predicted Target Genes and Pathway Enrichment Analysis

To further investigate the biological role of miR-196b-5p and miR-200b-3p, we performed bioinformatics research of their annotated targets. We identified 330 experimentally validated targets of both miRNAs (144 for miR-196b-5p and 186 for miR-200b-3p). The following four genes resulted as common targets of the two miRNAs: *BLC2*, *CDKN1B*, *TUBB*, *GATA6*.

A PANTHER ontology clustering of the identified targets was performed, based on pathway involvement (Table 4). Moreover, a pathway analysis using the Enrichr web tool was performed, based on KEGG pathways involvement (Table 5). 

The three most represented PANTHER pathways were the following: “insulin/IGF pathway-mitogen activated protein kinase kinase/MAP kinase cascade” (*p*-value 2.84 × 10^−2^ and fold enrichment 9.84), “Ras pathway” (*p*-value 4.58 × 10^−6^ and fold enrichment 9.76), and “insulin/IGF pathway-protein kinase B signaling cascade”( *p*-value 9.18 × 10^−3^ and fold enrichment 9.22) (Table 4).

The most 10 represented KEGG pathways are reported in Table 5: the combination score was used to sort these results. Especially the classes “colorectal cancer” (combined score 321, 34 and overlap genes 16/86), “prostate cancer” (combined score 309.67 and overlap genes 17/97), and “Neurotrophin signaling pathway” (combined score 257.80 and overlap genes 18/119) showed the best ranking (Table 5).

Regarding GO annotation enrichment analysis, the most represented molecular function classes were related to “binding” (259/305 annotated genes in GO:0005488 “binding” class) and “kinase activity” (31/305 annotated genes in GO:0016301) (Supplementary Table S3).

Enrichment analysis for biological processes predicted that the most represented classes were “biological regulation” (210/305 annotated genes in GO:0065007), and “macromolecule metabolic process” (170/305 annotated genes in GO:0043170) (Supplementary Table S3).

### 3.6. Genes Differentially Expressed in Adult vs. Childhood MBs According to GEO Datasets

The results of downregulated genes in adult MBs towards childhood MBs according to the Geo2R analyses are reported in Table 6. In the GSE49243 dataset (71 MBs, 26 children and 45 adults), a total of 404 genes resulted downregulated in adult MBs and 10 of these were targeted by miR-196b-5p or miR-200b-3p (Table 6). In the GSE41842 dataset (19 MBs, 14 children and 5 adults), a total of 105 genes resulted downregulated in adult MBs and 4 of these were targeted by miR-196b-5p or miR-200b-3p. In the GSE21140 dataset (102 MBs, 89 children and 13 adults), a total of 622 genes resulted downregulated in adult MBs and 13 of these were targeted by miR-196b-5p or miR-200b-3p. Two genes found downregulated in adult MBs and targeted by miR-196b-5p or miR-200b-3p (*IGF2BP3* and *MYB*) were common to two of three datasets (GSE49243 and GSE21140).

### 3.7. PPI Network of miR-196b-5p and miR-200b-3p Targets

**Figure 5:**
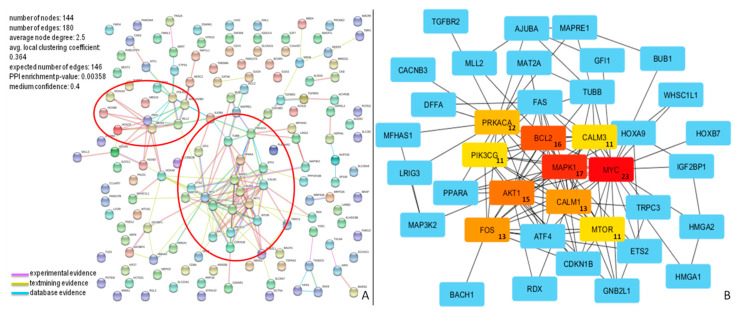
Protein–protein interaction (PPI) network for miR-196b-5p targets. (**A**) Proteins in the network are represented as colored points (“nodes”), a colored link represents each protein interaction (pink if experimental evidence, light green if text mining evidence, or light blue if database evidence, see Material and Methods section). Red ellipses highlight dense clusters; (**B**) The top ten nodes with a high degree in PPI of miR-196b-5p targets are represented in red, orange, or yellow boxes. The numbers inside the boxes represent the number of protein connections (degree) of each node.

**Figure 6:**
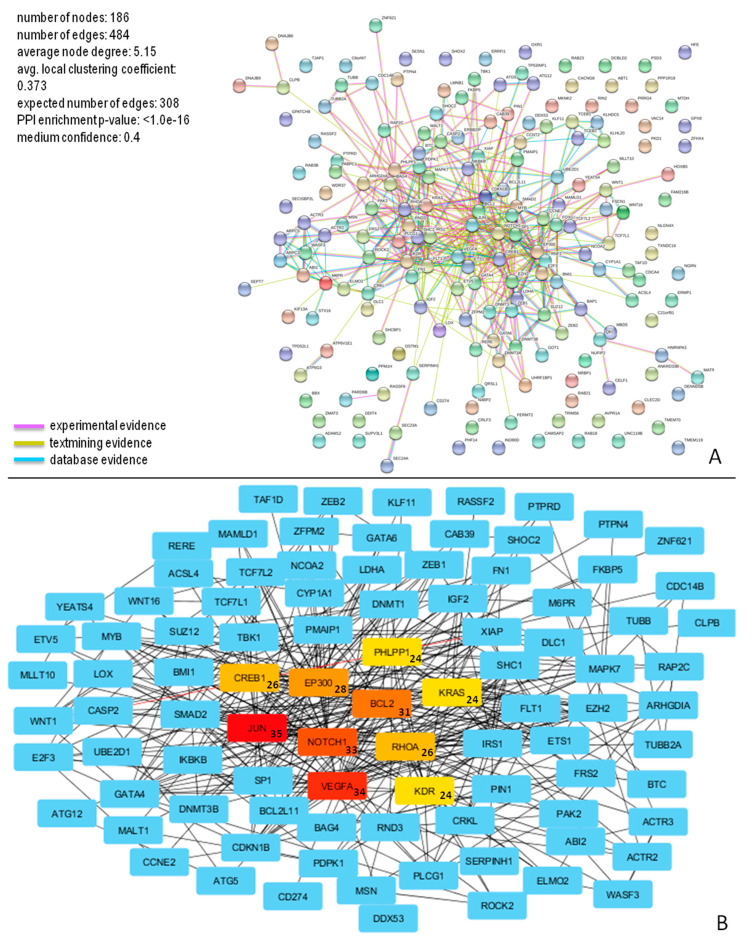
PPI network for miR-200b-3p targets. (**A**) Proteins in the network are represented as colored points (“nodes”), a colored link represents each protein interaction (pink if experimental evidence, light green if text mining evidence, or light blue if database evidence, see Material and Methods section); (**B**) The top ten nodes with a high degree in PPI of miR-200b-3p targets are represented in red, orange, or yellow boxes. The numbers inside the boxes represent the number of protein connections (degree) of each node.

The analysis of biological processes showed that the most represented GO classes for miR-196b-5p were related to “negative regulation of biological process” (GO:0048519), “negative regulation of cellular process” (GO:0048523), or “negative regulation of cellular metabolic process” (GO:0031324) (Supplementary Table S3). The results of in silico analysis show the possible biological implications of miR-196b-5p and miR-200b-3p. For each one of these two miRNAs, potential targets and pathways involved were studied (Figure 5 and Figure 6).

The molecular interactions of miR-196b-5p and miR-200b-3p are represented in Figure 5A and Figure 6A, respectively.

The network of miR-196b-5p consisted of 144 nodes and 180 edges, and showed significantly more interactions than expected, as indicated by STRING v10.5 analysis. Proteins in the network have more interactions among themselves than would be expected for a random set of proteins of similar size, suggesting that these targets are at least partially biologically connected. The top ten nodes with the highest degree values (*MYC*, *MAPK1*, *BCL2*, *AKT1*, *FOS*, *CALM1*, *PRKACA*, *CALM3*, *PIK3CG*, and *MTOR*) and their connections, are shown in Figure 5B.

Concerning miR-200b-3p, the analysis of biological processes showed that the most represented GO classes were related to “biological regulation” (GO:0065007), “macromolecule metabolic process” (GO:0044260), or “response to stimulus” (GO:0050896) (Supplementary Table S3).

This network consisted of 186 nodes and 484 edges, and showed significantly more interactions than expected, as indicated by STRING v10.5 analysis. These proteins also have more interactions among themselves than would be expected for a random set of proteins of similar size, suggesting that these targets are biologically connected. The top ten nodes with the highest degree values (*JUN*, *VEGFA*, *NOTCH1*, *BCL2*, *EP300*, *RHOA*, *CREB1*, *KDR*, *PHLPP1*, and *KRAS*) and their connections, are shown in Figure 6B.

## 4. Discussion

Medulloblastoma is a highly malignant brain tumor that occurs predominantly in children.

The WHO 2016 update presented a modular and integrated approach to the diagnosis of medulloblastoma, merging molecular and histological classification [12]. Accordingly, MBs are now classified into the following four categories that are associated with peculiar prognostic features: WNT-activated with classic or LCA (very rare) histology; SHH-activated (wild-type *TP53*) with classic, D/N, or LCA histology; SHH-activated (mutant *TP53*) with classic, LCA or D/N (very rare) histology; non-WNT/non-SHH group with classic or LCA histology (Group 3 and Group 4) [12,13].

In adults, MBs of the SHH molecular subgroup are the most represented and have an outcome similar to that of children [57]. Other tumors among adults are mainly diagnosed as WNT or “non-WNT/non-SHH, group 4”, while “non-WNT/non-SHH, group 3” are infrequent [15,57,58]. Although similar molecular profiles exist in adult and childhood MB, they occur with different frequencies and present a distinct genetic landscape and prognostic behavior [8,57,59].

The continued search for new biomarkers is paramount to improve diagnostic classification schemes further and to refine risk stratification [60].

MiRNAs represent ideal candidates for new targeted therapeutic approaches: the use of specific antisense (small interfering RNA, siRNA) to modulate overexpressed miRNAs or the replacement of downregulated miRNAs, have been approved in clinical trials and are being utilized in the clinical practice for several diseases [61,62].

Many studies have found the altered microRNA expression in various human tumors, including MB [18,19,20,21,22,23,24,25,26,27,28,29,30,31,32,33,34,35,36,37,38,39,40,41,42,43,44]. To date, studies have compared microRNAs expression between neoplastic and healthy tissue in adult or childhood MB, and some of them did not discriminate adult from childhood MBs [18,27,43,44]. In the present study we have compared miRNAs expression directly in adult towards childhood MBs. As previously reported, the relevance of “non-neoplastic” reference is crucial for determining the microRNAs tumoral expression. In fact, different control specimens (e.g., non-neoplastic cerebellum, commercial normal cerebellum RNA, and non-neoplastic specimens adjacent to the tumor) can lead to different data in miRNAs values, not only in MBs [18,27] but also in other tumors, such as gliomas [63]. A direct comparison of adult and childhood MBs provides further information about miRNAs profile, without being affected by the choice non-neoplastic reference.

This study investigates the miRNA expression profile of adult and childhood MBs. Interestingly, we found that some miRNAs (miR-9, miR-10b, miR-135a, miR-135b, miR-153, and miR-100) that were found deregulated in MB as compared with “healthy cerebellum” [18,27] were also differentially expressed in adult and childhood MB tissue, but with a lower “magnitude” observed, as compared with non-neoplastic tissue. At the same time, some miRNAs, found with a significantly different expression in MBs as compared with non-neoplastic specimens (e.g., miR-17, miR-99a, and miR-125) [18,27] are not differently expressed in adult and childhood MBs. This evidence leads to hypothesize that these miRNAs can be involved in MB development, but not in the different biological behavior of adult and childhood MBs.

Our data show that two microRNAs, miR-196b-5p and miR-200b-3p, are significantly overexpressed in adult MB as compared with childhood MB. Of interest, miR-196a-5p, which differs from the mature sequence of miR-196b-5p by only one nucleotide, but has a different chromosomal locus, was also downregulated, albeit to a low degree, in the adult group.

MiR-196b-5p is located on chromosome 7p15.2, in a highly conserved region near the *HOX* genes family [64].

The oncogenic role of miR-196b-5p has been reported in other malignant tumors such as glioblastoma [65], leukemia [66], gastric cancer [67], pancreatic cancer [68], and colorectal cancer [69,70,71].

Among the reported effects of dysregulated miR-196b-5p, the induction of cell proliferation, migration, and invasion have been demonstrated [65,72].

It has been established that miR-196b-5p regulates leukemias by inhibiting cell differentiation and apoptosis while promoting cell proliferation [73]. At the same time, miR-196b has also been found to suppress the expression of c-myc, a key regulator of cell proliferation and the anti-apoptotic factor Bcl-2 [74].

miR-200b-3p, located on chromosome 1p36.33, is known for its downregulation and anti-oncogenic role in many cancers such as colorectal [75] and breast cancers [76,77].

Lv et al. demonstrated that miR-200b-3p was significantly downregulated in metastatic colorectal cancer, with the concomitant upregulation of c-Myc and PRDX2, suggesting the importance of the c-Myc/miR-200b/PRDX2 axis in colorectal cancer progression and the development of distant metastases [75]. Some studies reported that miR-200b-3p acted as a tumor suppressor in breast cancer, inhibiting migration and invasion [78,79], suppressing proliferation, and inducing apoptosis [76,80,81,82].

Nevertheless, the role of miR-200b-3p is still controversial; in fact, it also has been reported as an oncomiR and not as oncosuppressor. For example, miR-200b-3p has been observed upregulated in oral squamous cell carcinoma (OSCC), as well as in plasma of patients with OSCC [83]. In contrast, miR-200b-3p was significantly downregulated in OSCC patients after surgery, suggesting that the detection of circulating miR-200b-3p could be a potential diagnostic biomarker in OSCC [83].

In lung cancer, two meta-analyses showed that in 20 [84] and 26 [85] published microRNAs profiling studies, miR-200b-3p was identified as consistently upregulated. In another study, it has been demonstrated that the combination of four miRNAs (miR-21, miR-486, miR-375, and miR-200b) was sufficient to distinguish between non-small cell lung cancer (NSCLC) patients and healthy controls (81% sensitivity and 92% specificity) [86].

Combing the miR-196b-5p and miR-200b-3p results, we retrieved 330 experimentally validated targets. Functional enrichment for molecular function analysis predicted that the most represented classes were related to “binding” and “kinase activity”; whereas the enrichment analysis for biological processes predicted that the most represented classes were related to “biological regulation” and “macromolecule metabolic process”. We clustered these results considering pathway involvement and the three most overrepresented PANTHER pathways included “insulin/IGF pathway-mitogen activated protein kinase kinase/MAP kinase cascade”, “Ras pathway”, and “insulin/IGF pathway-protein kinase B signaling cascade”. It has previously been demonstrated that all these pathways play a role in MB pathogenesis [87,88,89,90].

MAPK signaling and RAS pathways are altered in many tumors, mainly for the presence of oncogenic mutations [91]. In childhood medulloblastoma the RAS/MAPK pathway is often overexpressed [92] and RAS inhibitors were proposed as potentially therapeutic agents for metastatic disease [92] or for specific molecular MB subgroup [93]. Smo inhibitors, such as Sonidegib (LDE225) [94] and Vismodegib (GDC-0449) [95,96,97], showed a good response in SHH MB treatment, both in adults and children, supporting their possible combination with conventional chemotherapies [98]. However, constitutive RAS/MAPK activation was reported to induce resistance to the Smo inhibitor Vismodegib. In fact, tumors with activated RAS/MAPK cascade can evolve and escape from Shh signaling control [99]. In MB, RAS/MAPK pathway activation depends on alternative mechanisms, since activating mutations seem to be rare events, as observed in this study (data not shown) and reported by Gilbertson et al. [100]. In our analysis, miR-196b-5p and miR-200b-3b are overexpressed in adult MBs as compared with childhood MBs and seem to negatively control several genes involved in the RAS/MAPK cascade (e.g., *MAPK1* and *KRAS*). Further studies must be addressed to validate these results and to understand how these pathways really act in adult MB, considering both the potential role of RAS/MAPK genes as therapeutic targets and the RAS/MAPK activation as the principal actor in drug resistance.

The most significantly enriched pathways (KEGG classes) included the following: “colorectal cancer”, “prostate cancer” and “neurotrophin signaling pathway”. We found that many target genes are common to different enriched classes, for example, the aforementioned *MAPK1* (targeted by miR-196b-5p) and *KRAS* (targeted by miR-200b-3p) are common targets in all the most represented pathways. *BCL2*, a common target of mir-196b-5p and miR-200b-3p implicated in the apoptosis cascade, is also involved in “colorectal cancer”, “prostate cancer”, and “neurotrophin signaling pathway”. BCL2 expression has been previously investigated in MB and it was found to be IHC positive in patients with tumor cell undifferentiation, aged less than three or more than fifteen years [101]. Schüller and colleagues showed that patients with classical BCL2 positive MBs had a poorer outcome than those with classical BLC2 negative tumors [101]. In light of these data and those obtained in the present study, it would be interesting to evaluate the expression of BLC2 protein by IHC analysis in a vast cohort of adult MBs.

As considered in a previous study, target genes which could have a prognostic impact on a MB subgroup, were not necessarily highly expressed in the particular subgroup as compared with others. These results, together with our considerations, indicate that the choice of therapeutic targets and strategies should not consider only the genes that are highly expressed [93].

According to the results obtained using GEO datasets, the genes found deregulated in adult MBs towards childhood MBs, and targeted by miR-196b-5p and miR-200b-3p are frequently found to play a role in “cancer pathways”, “apoptosis”, “ras signaling pathway”, and “PI3K-Akt signaling pathway” among others. Intriguingly, two genes targeted by miR-196b-5p and miR-200b-3p were found downregulated in adult MBs in the two datasets with the higher number of cases (GSE49243 and GSE21140): (i) *IGF2BP3* (insulin-like growth factor-2 mRNA-binding protein 3) and (ii) *Myb* (c-myb proto-oncogene). The fact that these target are usually upregulated in several tumors [102,103], while it seems to be downregulated in adult MBs as compared with childhood MBs, endorses the evidence that “medulloblastoma in adults are not just big kids” [104]. Intriguingly, deficient IGF-2 tumor cells were more sensitive to chemotherapy-induced apoptosis [105]. In high-risk medulloblastoma patients, survival can be improved by the addition of chemotherapy [106], but nowadays other markers for predicting response are needed [106,107]. Trying to associate low IGF2BP3 levels with response to chemotherapy could help to identify a subset of patients more responsive to the treatment.

Considering annotated targets of miR-196b-5p and miR-200b-3p separately (144 and 186 genes, respectively), our analysis showed potential extensive gene networks, consistent with the concept that a single miRNA controls different biological processes by targeting multiple related genes. In particular, 10 target genes of miR-196b-5p (*MYC*, *MAPK1*, *BCL2*, *AKT1*, *FOS*, *CALM1*, *PRKACA*, *CALM3*, *PIK3CG*, and *MTOR*) and 10 of miR-200b-3p (*JUN*, *VEGFA*, *NOTCH1*, *BCL2*, *EP300*, *RHOA*, *CREB1*, *KDR*, *PHLPP1*, and *KRAS*) had the highest number of protein–protein interactions, and it is not surprising that they appeared to act in most overrepresented pathways found in our analysis.

Our data also showed that the AD-SHH subgroup is characterized by overexpression of miR-196b-5p as compared with AD-WNT and AD non-WNT/non-SHH cases and by the absence of miR-183-5p.

Further experiments focused on expression of targets that were found deregulated, and in silico analysis would be performed. In fact, detecting different patterns of protein expression in adult MBs towards childhood MBs could help in discovering new putative therapeutic or prognostic targets. Due to the lack of adult MB cases, no specific therapies have been established, and treatment modalities have been adapted from pediatric protocols. Surgery and postoperative craniospinal radiotherapy remain the mainstay treatments for this disease.

Several studies have shown that high-risk patients should be treated with adjuvant chemotherapy, but its role remains unclear in standard- or average-risk adult MB [108].

In the work of Zhao et al., adjuvant chemotherapy did not correlate with prognosis in high-risk patients or average-risk patients, indicating that the current chemotherapy protocol needs to be optimized for adult MB [109].

Considering the controversial efficacy of standard treatments in adult MB, it is crucial to discover new agents and therapeutic approaches.

The molecular classification of MB has allowed improved risk stratification and molecularly informed clinical trials; a similar approach would be beneficial for adult MBs [104,109,110].

MiRNAs represent attractive targets which manipulate several cellular functions at the same time. Inhibition of overexpressed oncogenic miRNAs or substitution of tumor-suppressive miRNAs could become a robust strategy for cancer therapy [111]. Consequently, as summarized by Ajay et al., there has been a rapid growth in patent applications in the last few years; the annual number of U.S. and European published patent applications and issued patents related to miRNAs reaching close to 500 [61]. As reported in the detailed review of Chakraborty et al., to date, approximately 20 clinical trials have been initiated using miRNA- and siRNA-based therapeutics [62].

Only a very few studies have investigated miRNA expression in medulloblastomas of both children and adult patients [43]. In our study, miRNAs expression analysis has been performed directly comparing adult vs. childhood MBs, without using a non-neoplastic reference. As previously demonstrated, the choice of appropriate and consistent controls is crucial for obtaining robust data. In fact, different control groups could obtain different results in miRNA analysis [63]. Then, the primary focus of our research is the characterization of miRNA expression in the rare adult MB, and we demonstrate, for the first time, that adult and childhood MBs have different miRNA expression profiles.

In this study, we observed that miR-196b-5p and miR-200b-3p are significantly overexpressed in MBs of adults as compared with those of children. Here, these miRNAs that resulted in differential expression between the two groups were further studied, with in silico analysis, to investigate their biological roles. This data could the basis for future in-depth analysis to fully elucidate the implications of miR-196b-5p and miR-200b-3p dysregulation, for example, in different medulloblastoma behavior in adult or child subjects.

## Figures and Tables

**Table 1:** GEO datasets selected for the analysis.

GEO Accession Number	Number Samples (Children/Adult)	Platform
GSE41842	19 (15/4)	Affymetrix Human Gene 1.0 ST Array (transcript (gene) version)
GSE49243	71 (26/45)	Affymetrix Human Genome U133 Plus 2.0 Array
GSE21140	102 (89/13)	Affymetrix Human Exon 1.0 ST Array (transcript (gene) version]

**Table 2:** Patients characteristics, histological, and molecular subgroup distribution.

	AD		CH	
*Mean age*	30.48		6.58	
Range	16–66		1–15	
*Sex ratio M:F*	14:7		12:7	
**Histological subtype**				
	No.	%	No.	%
*CL*	9	42.86%	12	63.16%
*D/N*	9	42.86%	4	21.05%
*LCA*	3	14.28%	3	15.79%
**Molecular subtype**				
	No.	%	No.	%
*WNT*	4	19.05%	4	21.05%
*SHH*	7	33.33%	3	15.79%
*non-WNT/non-SHH*	10	47.62%	12	63.16%

AD, adult; CH, children; CL, classic histological subtype; LCA, large cells anaplastic histological subtype; D/N, desmoplastic/nodular histological subtype; WNT, WNT molecular subgroup; SHH, SHH molecular subgroup; non-WNT/non-SHH, non-WNT/non-SHH molecular subgroup.

**Table 3:** MicroRNAs differentially expressed in the adult versus the childhood medulloblastoma training sets.

**miRNA Upregulated**					
	**F.C.**		**F.C.**		**F.C.**
miR-196b-5p	77.891	miR-221-3p	3.613	miR-485-3p	2.194
miR-200b-3p	11.904	miR-376b-3p	3.613	miR-888-5p	2.134
miR-193a-3p	10.080	miR-299-5p	3.539	miR-296-5p	2.119
miR-376a-3p	8.187	let-7f-5p	3.515	miR-29b-3p	2.119
miR-451a	8.075	miR-539-5p	3.490	miR-345-5p	2.119
miR-452-5p	7.534	miR-346	3.348	let-7b-5p	2.104
miR-190a	7.328	miR-223-3p	3.302	miR-302a-3p	2.090
miR-590-5p	7.127	miR-9-3p	3.302	miR-411-5p	2.075
miR-625-3p	6.513	let-7i-5p	3.212	miR-20a-5p	2.061
miR-27a-3p	6.379	miR-24-3p	3.146	miR-140-5p	2.047
miR-142-3p	6.035	miR-146b-5p	3.124	miR-335-5p	2.047
miR-374b-5p	5.911	miR-153	3.102	miR-98-5p	2.047
miR-27b-3p	5.670	miR-28-5p	2.875	miR-660-5p	2.033
miR-376c-3p	5.515	miR-127-3p	2.855		
miR-21-3p	5.364	miR-136-5p	2.777		
miR-100-5p	5.290	miR-582-5p	2.720		
miR-31-5p	5.075	miR-92b-3p	2.701		
miR-374a-5p	5.040	miR-20b-3p	2.609		
miR-365a-3p	4.835	miR-195-5p	2.555		
miR-193a-5p	4.702	miR-323a-3p	2.434		
miR-135a-5p	4.670	let-7d-3p	2.401		
miR-363-3p	4.574	miR-627	2.351		
miR-137	4.511	let-7d-5p	2.303		
miR-671-5p	4.357	miR-424-5p	2.287		
miR-33a-5p	3.954	miR-497-5p	2.287		
**miRNA Downregulated**					
	**F.C.**		**F.C.**		**F.C.**
miR-183-5p	−70.356	miR-665	−3.379	miR-184	−2.066
miR-642a-5p	−53.320	miR-449a	−3.356	miR-339-5p	−2.051
miR-182-5p	−43.010	miR-455-5p	−3.356	miR-133b	−2.037
miR-196a-5p	−33.281	miR-873-5p	−3.287		
miR-96-5p	−29.377	miR-181a-3p	−3.197		
miR-206	−20.629	miR-890	−3.109		
miR-760	−10.979	miR-135b-5p	−2.983		
miR-181b-5p	−10.827	miR-181d	−2.764		
miR-877-5p	−8.554	miR-595	−2.633		
miR-124-3p	−7.344	miR-662	−2.615		
miR-506-3p	−5.965	miR-10b-5p	−2.561		
miR-181a-5p	−5.843	miR-211-5p	−2.508		
miR-490-3p	−4.845	miR-187-5p	−2.457		
miR-373-5p	−4.616	miR-187-3p	−2.423		
miR-302c-5p	−4.277	miR-454-3p	−2.356		
miR-542-5p	−4.277	miR-301a-3p	−2.308		
miR-200c-3p	−4.074	miR-421	−2.260		
miR-203a	−3.991	miR-155-5p	−2.245		
miR-501-5p	−3.855	miR-324-3p	−2.245		
miR-488-3p	−3.802	miR-520c-3p	−2.229		
miR-138-5p	−3.749	miR-34b-3p	−2.214		
miR-324-5p	−3.698	miR-20b-5p	−2.199		
miR-141-3p	−3.672	miR-765	−2.168		
miR-29b-2-5p	−3.622	miR-126-5p	−2.139		
miR-628-3p	−3.523	miR-152	−2.139		

F.C., fold changes.

**Table 4:** PANTHER overrepresentation pathway analysis of miR-196b-5p and miR-200b-3p targets.

PANTHER Pathways	Number of Genes					
	Reference List *	Target List ^†^	Fold Enrichment Scores	*p*-Value	miR-196b-5p Target Genes	miR-200b-3p Target Genes
Insulin/IGF pathway-mitogen activated protein kinase kinase/MAP kinase cascade	32	5	9.84	2.84 × 10^−2^	*MAPK1*, *FOS*	*IGF2*, *M6PR*, *IRS1*
Ras Pathway	71	11	9.76	4.58 × 10^−6^	*MAPK1*, *AKT1*, *PIK3CG*, *RGL2*	*KRAS*, *RHOA*, *SHC1*, *JUN*, *ETS1*, *PDPK1*, *PAK2*
Insulin/IGF pathway-protein kinase B signaling cascade	41	6	9.22	9.18 × 10^−3^	*AKT1*, *PIK3CG*	*IGF2*, *M6PR*, *PDPK1*, *IRS1*
Interleukin signaling pathway	83	12	9.11	2.28 × 10^−6^	*MAPK1*, *AKT1*, *MTOR*, *MYC*, *FOS*, *CDKN1B*	*IKBKB*, *SHC1*, *MKNK2*, *PDPK1*, *IRS1*, *CDKN1B*, *MAPK7*
VEGF signaling pathway	65	9	8.72	2.09 × 10^−4^	*MAPK1*, *AKT1*, *PIK3CG*, *PRKACA*	*KDR*, *PKD1*, *ETS1*, *VEGFA*, *PLCG1*
Apoptosis signaling pathway	119	16	8.47	2.52 × 10^−8^	*MAPK1*, *FAS*, *AKT1*, *PIK3CG*, *ATF4*, *PRKACA*, *BCL2*, *FOS*	*IKBKB*, *M6PR*, *CREB1*, *KLHL20*, *JUN*, *BAG4*, *XIAP*, *BCL2*, *BCL2L11*
B cell activation	65	8	7.75	1.88 × 10^−3^	*MAPK1*, *MAP3K2*, *PIK3CG*, *CALM1*, *FOS*	*IKBKB*, *JUN*, *RHOA*
p53 pathway feedback loops 2	51	6	7.41	2.98 × 10^−2^	*AKT1*, *PIK3CG*, *MYC*	*KRAS*, *E2F3*, *PDPK1*
Angiogenesis	161	18	7.04	3.36 × 10^−8^	*MAPK1*, *AKT1*, *PIK3CG*, *PRKACA*, *FOS*	*PKD1*, *RHOA*, *JUN*, *FRS2*, *CRKL*, *KDR*, *SHC1*, *ETS1*, *WNT1*, *TCF7L2*, *PAK2*, *VEGFA*, *PLCG1*
CCKR signaling map	162	17	7.00	3.71 × 10^−8^	*MAPK1*, *AKT1*, *PRKACA*, *CALM1*, *MYC*, *BCL2*, *FOS*	*CREB1*, *PKD1*, *RHOA*, *SHC1*, *JUN*, *PDPK1*, *IRS1*, *MAPK7*, *BCL2*, *SP1*, *PLCG1*
PDGF signaling pathway	144	16	7.00	3.73 × 10^−7^	*MAPK1*, *MAP3K2*, *MTOR*, *PIK3CG*, *MYC*, *PRKACA*, *FOS*	*IKBKB*, *DLC1*, *SHC1*, *JUN*, *ETS1*, *MKNK2*, *PDPK1*, *MAPK7*, *PLCG1*
T cell activation	85	9	6.67	1.77 × 10^−3^	*MAPK1*, *AKT1*, *PIK3CG*, *CALM1*, *FOS*	*IKBKB*, *JUN*, *PAK2*, *PLCG1*
p53 pathway	88	8	5.73	1.56 × 10^−2^	*FAS*, *AKT1*, *PIK3CG*	*E2F3*, *PMAIP1*, *EP300*, *ZMAT3*, *PDPK1*
Integrin signalling pathway	162	12	5.44	7.61 × 10^−5^	*MAPK1*, *MAP3K2*, *LAMB2*, *PIK3CG*	*CRKL*, *FN1*, *PTPN12*, *RND3*, *RHOA*, *SHC1*, *ARPC3*, *RAP2C*
Gonadotropin-releasing hormone receptor pathway	227	18	5.27	1.14 × 10^−6^	*MAPK1*, *MAP3K2*, *LHX2*, *ACVR2B*, *AKT1*, *TGFBR3*, *PBX1*, *PRKACA*, *FOS*	*CREB1*, *GATA4*, *ZEB1*, *SMAD2*, *EP300*, *JUN*, *TCF7L1*, *SP1*, *PPARA*
EGF receptor signaling pathway	135	11	5.13	2.20 × 10^−3^	*MAPK1*, *MAP3K2*, *AKT1*, *PIK3CG*, *PRKACA*	*BTC*, *PKD1*, *RHOA*, *SHC1*, *MAPK7*, *PLCG1*
FGF signaling pathway	120	9	4.73	2.44 × 10^−2^	*MAPK1*, *MAP3K2*, *AKT1*, *PIK3CG*, *PRKACA*	*FRS2*, *RHOA*, *SHC1*, *PLCG1*
Inflammation mediated by chemokine and cytokine signaling pathway	230	13	3.56	1.59 × 10^−2^	*MAPK1*, *AKT1*, *PRKACA*, *PIK3CG*	*IKBKB*, *KRAS*, *RHOA, JUN*, *PLCG1*, *SHC1*, *ARPC3*, *PDPK1*, *PAK2*

Overrepresented pathways are shown following fold enrichment scores. *P*-values are determined by binomial statistics with Bonferroni correction and a *p*-value cutoff of 0.05 was considered. * Number of genes in the reference list that map to this specific PANTHER pathway and ^†^ Number of genes in the target genes list that map to this specific PANTHER pathway. Underlined genes are common targets of miR-196b-5p and miR-200b-3p.

**Table 5:** Enrichr pathway analysis of miR-196b-5p and miR-200b-3p targets. The top 10 KEGG classes are reported.

Index	Name	Overlap	miR-196b-5p Target Genes	miR-200b-3p Target Genes
1	Colorectal cancer	16/86	*FOS*, *MTOR*, *TGFBR2*, *MSH6*, *MYC*, *BCL2*, *AKT1*, *MAPK1*	*SMAD2*, *TCF7L2*, *JUN*, *TCF7L1*, *RHOA*, *BCL2L11*, *BCL2*, *KRAS*, *PMAIP1*
2	Prostate cancer	17/97	*CDKN1B*, *MTOR*, *IKBKB*, *BCL2*, *AKT1*, *MAPK1*, *ATF4*	*TCF7L2*, *TCF7L1*, *CDKN1B*, *PDPK1*, *ETV5*, *CREB1*, *ZEB1*, *CCNE2*, *BCL2*, *EP300*, *E2F3*, *KRAS*
3	Neurotrophin signaling pathway	18/119	*BLC2*, *AKT1*, *MAPK1*, *CALM3*, *CALM1*, *ATF4*	*JUN*, *SHC1*, *IRS1*, *PDPK1*, *FRS2*, *RHOA*, *CRKL*, *IKBKB*, *MAPK7*, *ARHGDIA*, *BCL2*, *KRAS*, *PLCG1*
4	MicroRNAs in cancer	32/299	*CDKN1B*, *MYC*, *IGFBP1*, *MAPK1*, *RDX*, *HMGA2*, *MTOR*, *BLC2*	*DNMT1*, *CDKN1B*, *NOTCH1*, *IRS1*, *SHC1*, *BMI1*, *CRKL*, *IKBKB*, *BCL2L11*, *MAPK7*, *DNMT3B*, *EP300*, *E2F3*, *PLCG1*, *DNMT3A*, *RHOA*, *VEGFA*, *ZEB2*, *ZEB1*, *CCNE2*, *DDIT4*, *FSCN1*, *BCL2*, *KRAS*, *ZFPM2*, *EZH2*
5	Proteoglycans in cancer	21/201	*RDX*, *MTOR*, *MYC*, *AKT1*, *MAPK1*, *FAS*, *PRKACA*	*SMAD2*, *PDPK1*, *ROCK2*, *IGF2*, *FN1*, *MSN*, *FRS2*, *WNT16*, *RHOA*, *VEGFA*, *KDR*, *KRAS*, *PLCG1*, *WNT1*
6	ErbB signaling pathway	12/85	*CDKN1B*, *MYC*, *AKT1*, *MAPK1*, *MTOR*	*BTC*, *JUN*, *CDKN1B*, *SHC1*, *KRAS*, *PLCG1*, *PAK2*, *CRKL*
7	Pathways in cancer	39/530	*CDKN1B*, *MYC*, *AKT1, MAPK1*, *PRKACA*, *LAMB2*, *FOS*, *MTOR*, *TGFBR2*, *MSH6*, *BCL2*, *FAS*, *CALM3*, *CALM1*	*CDKN1B*, *NOTCH1*, *BCL2L11*, *KRAS*, *CRKL*, *IKBKB*, *ROCK2*, *XIAP*, *ETS1*, *EP300*, *PMAIP1*, *E2F3*, *PLCG1*, *ELOC*, *WNT1*, *SMAD2*, *TCF7L2*, *JUN*, *TCF7L1*, *FN1*, *IGF2*, *WNT16*, *RHOA*, *VEGFA*, *CCNE2*, *SP1*, *BCL2*
8	HIF-1 signaling pathway	13/100	*CDKN1B*, *MTOR*, *BCL2*, *AKT1*, *MAPK1*, *ALDOA*	*FLT1*, *CDKN1B*, *VEGFA*, *LDHA*, *MKNK2*, *BCL2*, *EP300*, *PLCG1*, *ELOC*
9	Apoptosis	16/143	*DFFA*, *FOS*, *BCL2*, *AKT1*, *MAPK1*, *FAS*, *ATF4*	*JUN*, *PDPK1*, *XIAP*, *LMNB1*, *IKBKB*, *BCL2L11*, *BCL2*, *PMAIP1*, *CASP2*, *KRAS*
10	Renal cell carcinoma	10/69	*AKT1*, *MAPK1*	*JUN*, *EP300*, *KRAS*, *ELOC*, *ETS1*, *PAK2*, *CRKL*, *VEGFA*

The “KEGG 2019 Human” Enrichr web tool was used. Enrichment pathway data were sorted according to combined scores, as suggested by the Enrichr instructions. Overlap, ratio between number of genes in the target genes list that map to this specific KEGG class. Underlined genes are common targets of miR-196b-5p and miR-200b-3p.

**Table 6:** Target downregulated in adult MBs vs. childhood MBs, according to the GeoR2 analysis performed on three different GEO datasets.

DataSets	Number of Downregulated Genes in Ad vs. Ch Mbs	Downregulated Genes Targeted by miR-196b-5p or miR-200b-3p	KEGG 2019 Human Pathway
GSE49243	404	N = 10	
		*IGF2BP3*	-
		*ETS1*	**Pathways in cancer**, Renal cell carcinoma, **Human T-cell leukemia virus 1 infection**, **Ras signaling pathway**, Cellular senescence
		*PMAIP1*	**Pathways in cancer**, p53 signaling pathway, Colorectal cancer, **Apoptosis**, Viral carcinogenesis
		*ZFPM2*	MicroRNAs in cancer
		*LRIG3*	-
		*CD8A*	Primary immunodeficiency, Antigen processing and presentation, Hematopoietic cell lineage, T cell receptor signaling pathway, Cell adhesion molecules (CAMs)
		*OXR1*	-
		*FKBP5*	Estrogen signaling pathway
		*TPD52L1*	-
		*MYB*	**PI3K-Akt signaling pathway**
GSE41842	105	N = 4	
		*LMNB1*	**Apoptosis**
		*DCBLD2*	-
		*CTPS1*	Pyrimidine metabolism
		*E2F3*	Bladder cancer, Non-small cell lung cancer, Melanoma, Pancreatic cancer, Glioma, Chronic myeloid leukemia, Small cell lung cancer, Prostate cancer, Cell cycle, **Breast cancer**, Gastric cancer, Cushing syndrome, Hepatitis C, Cellular senescence, Hepatitis B, Hepatocellular carcinoma, Kaposi sarcoma-associated herpesvirus infection, Epstein-Barr virus infection, **Human T-cell leukemia virus 1 infection**, Human cytomegalovirus infection, **MicroRNAs in cancer**, **Pathways in cancer**
GSE21140	622	N = 13	
		*KCNJ2*	**Cholinergic synapse**, **Oxytocin signaling pathway**, Renin secretion, Gastric acid secretion
		*KDR*	**Fluid shear stress and atherosclerosis**, **MAPK signaling pathway**, **PI3K-Akt signaling pathway**, VEGF signaling pathway, Focal adhesion, Proteoglycans in cancer, Rap1 signaling pathway, **Ras signaling pathway**
		*IGF2BP3*	-
		*HERC2*	Ubiquitin mediated proteolysis
		*FOS*	**Cholinergic synapse**, **Fluid shear stress and atherosclerosis**, **Oxytocin signaling pathway**, **MAPK signaling pathway**, Amphetamine addiction, Prolactin signaling pathway, B cell receptor signaling pathway, Leishmaniasis, Pertussis, Salmonella infection, **Colorectal cancer**, Rheumatoid arthritis, Th1 and Th2 cell differentiation, IL-17 signaling pathway, Circadian entrainment, Choline metabolism in cancer, T cell receptor signaling pathway, Chagas disease (American trypanosomiasis), Toll-like receptor signaling pathway, Parathyroid hormone synthesis secretion and action, Th17 cell differentiation, TNF signaling pathway, Osteoclast differentiation, Relaxin signaling pathway, Dopaminergic synapse, Estrogen signaling pathway, Measles, **Apoptosis**, **Breast cancer**, Hepatitis B, Kaposi sarcoma-associated herpesvirus infection, Human immunodeficiency virus 1 infection, cAMP signaling pathway, **Human T-cell leukemia virus 1 infection**, **Pathways in cancer**
		*ARID5B*	Protein processing in endoplasmic reticulum
		*HSPA4L*	-
		*ZFHX4*	-
		*DLC1*	-
		*TRIM56*	-
		*MTUS1*	-
		*FOXG1*	FoxO signaling pathway
		*MYB*	**PI3K-Akt signaling pathway**

In bold, pathways common to more than one gene, and underlined the genes common to more datasets.

## Supplementary Materials

The following are available online at https://www.mdpi.com/2075-4418/10/5/265/s1, Table S1: Genes differently expressed in adult MBs vs. children MB, according to GEO datasets, Table S2: CTNNB1 mutations founded in adult and childhood medulloblastoma, Table S3: GO annotation for molecular functions and biological processes.

## References

[1] Rickert C.H., Paulus W. (2001). Epidemiology of central nervous system tumors in childhood and adolescence based on the new WHO classification. Childs Nerv. Syst..

[2] McNeil D.E., Cote T.R., Clegg L., Rorke L.B. (2002). Incidence and trends in pediatric malignancies medulloblastoma/primitive neuroectodermal tumor: A SEER update. Surveillance Epidemiology and End Results. Med. Pediatr. Oncol..

[3] Ostrom Q.T., de Blank P.M., Kruchko C., Petersen C.M., Liao P., Finlay J.L., Stearns D.S., Wolff J.E., Wolinsky Y., Letterio J.J. (2015). Alex’s Lemonade Stand Foundation Infant and Childhood Primary Brain and Central Nervous System Tumors Diagnosed in the United States in 2007–2011. Neuro Oncol..

[4] Smoll N.R., Drummond K.J. (2012). The incidence of medulloblastomas and primitive neurectodermal tumours in adults and children. J. Clin. Neurosci..

[5] Bloom H.J., Bessell E.M. (1990). Medulloblastoma in adults: A review of 47 patients treated between 1952 and 1981. Int. J. Radiat. Oncol. Biol. Phys..

[6] Wrensch M., Minn Y., Chew T., Bondy M., Berger M.S. (2002). Epidemiology of primary brain tumors: Current concepts and review of the literature. Neuro Oncol..

[7] Giordana M.T., Cavalla P., Dutto A., Borsotti L., Chio A., Schiffer D. (1997). Is medulloblastoma the same tumor in children and adults?. J. Neurooncol..

[8] Korshunov A., Remke M., Werft W., Benner A., Ryzhova M., Witt H., Sturm D., Wittmann A., Schottler A., Felsberg J. (2010). Adult and pediatric medulloblastomas are genetically distinct and require different algorithms for molecular risk stratification. J. Clin. Oncol..

[9] Rodriguez F.J., Eberhart C., O’Neill B.P., Slezak J., Burger P.C., Goldthwaite P., Wu W., Giannini C. (2007). Histopathologic grading of adult medulloblastomas. Cancer.

[10] Ang C., Hauerstock D., Guiot M.C., Kasymjanova G., Roberge D., Kavan P., Muanza T. (2008). Characteristics and outcomes of medulloblastoma in adults. Pediatr. Blood Cancer.

[11] Parsons D.W., Li M., Zhang X., Jones S., Leary R.J., Lin J.C., Boca S.M., Carter H., Samayoa J., Bettegowda C. (2011). The genetic landscape of the childhood cancer medulloblastoma. Science.

[12] Louis D.N., Perry A., Reifenberger G., von Deimling A., Figarella-Branger D., Cavenee W.K., Ohgaki H., Wiestler O.D., Kleihues P., Ellison D.W. (2016). The 2016 World Health Organization Classification of Tumors of the Central Nervous System: A summary. Acta Neuropathol..

[13] Archer T.C., Mahoney E.L., Pomeroy S.L. (2017). Medulloblastoma: Molecular Classification-Based Personal Therapeutics. Neurotherapeutics.

[14] Al-Halabi H., Nantel A., Klekner A., Guiot M.C., Albrecht S., Hauser P., Garami M., Bognar L., Kavan P., Gerges N. (2011). Preponderance of sonic hedgehog pathway activation characterizes adult medulloblastoma. Acta Neuropathol..

[15] Remke M., Hielscher T., Northcott P.A., Witt H., Ryzhova M., Wittmann A., Benner A., von Deimling A., Scheurlen W., Perry A. (2011). Adult medulloblastoma comprises three major molecular variants. J. Clin. Oncol..

[16] Esquela-Kerscher A., Slack F.J. (2006). Oncomirs—MicroRNAs with a role in cancer. Nat. Rev. Cancer.

[17] Volinia S., Calin G.A., Liu C.G., Ambs S., Cimmino A., Petrocca F., Visone R., Iorio M., Roldo C., Ferracin M. (2006). A microRNA expression signature of human solid tumors defines cancer gene targets. Proc. Natl. Acad. Sci. USA.

[18] Ferretti E., De Smaele E., Po A., Di Marcotullio L., Tosi E., Espinola M.S., Di Rocco C., Riccardi R., Giangaspero F., Farcomeni A. (2009). MicroRNA profiling in human medulloblastoma. Int. J. Cancer.

[19] Pang J.C., Kwok W.K., Chen Z., Ng H.K. (2009). Oncogenic role of microRNAs in brain tumors. Acta Neuropathol..

[20] Pierson J., Hostager B., Fan R., Vibhakar R. (2008). Regulation of cyclin dependent kinase 6 by microRNA 124 in medulloblastoma. J. Neurooncol..

[21] Northcott P.A., Fernandez L.A., Hagan J.P., Ellison D.W., Grajkowska W., Gillespie Y., Grundy R., Van Meter T., Rutka J.T., Croce C.M. (2009). The miR-17/92 polycistron is up-regulated in sonic hedgehog-driven medulloblastomas and induced by N-myc in sonic hedgehog-treated cerebellar neural precursors. Cancer Res..

[22] Weeraratne S.D., Amani V., Teider N., Pierre-Francois J., Winter D., Kye M.J., Sengupta S., Archer T., Remke M., Bai A.H. (2012). Pleiotropic effects of miR-183~96~182 converge to regulate cell survival, proliferation and migration in medulloblastoma. Acta Neuropathol..

[23] Uziel T., Karginov F.V., Xie S., Parker J.S., Wang Y.D., Gajjar A., He L., Ellison D., Gilbertson R.J., Hannon G. (2009). The miR-17~92 cluster collaborates with the Sonic Hedgehog pathway in medulloblastoma. Proc. Natl. Acad. Sci. USA.

[24] Murphy B.L., Obad S., Bihannic L., Ayrault O., Zindy F., Kauppinen S., Roussel M.F. (2013). Silencing of the miR-17~92 cluster family inhibits medulloblastoma progression. Cancer Res..

[25] Bai A.H., Milde T., Remke M., Rolli C.G., Hielscher T., Cho Y.J., Kool M., Northcott P.A., Jugold M., Bazhin A.V. (2012). MicroRNA-182 promotes leptomeningeal spread of non-sonic hedgehog-medulloblastoma. Acta Neuropathol..

[26] Grunder E., D’Ambrosio R., Fiaschetti G., Abela L., Arcaro A., Zuzak T., Ohgaki H., Lv S.Q., Shalaby T., Grotzer M. (2011). MicroRNA-21 suppression impedes medulloblastoma cell migration. Eur. J. Cancer.

[27] Liu W., Gong Y.H., Chao T.F., Peng X.Z., Yuan J.G., Ma Z.Y., Jia G., Zhao J.Z. (2009). Identification of differentially expressed microRNAs by microarray: A possible role for microRNAs gene in medulloblastomas. Chin. Med. J. (Engl.).

[28] Garzia L., Andolfo I., Cusanelli E., Marino N., Petrosino G., De Martino D., Esposito V., Galeone A., Navas L., Esposito S. (2009). MicroRNA-199b-5p impairs cancer stem cells through negative regulation of HES1 in medulloblastoma. PLoS ONE.

[29] Li K.K., Pang J.C., Ching A.K., Wong C.K., Kong X., Wang Y., Zhou L., Chen Z., Ng H.K. (2009). miR-124 is frequently down-regulated in medulloblastoma and is a negative regulator of SLC16A1. Hum. Pathol..

[30] Venkataraman S., Alimova I., Fan R., Harris P., Foreman N., Vibhakar R. (2010). MicroRNA 128a increases intracellular ROS level by targeting Bmi-1 and inhibits medulloblastoma cancer cell growth by promoting senescence. PLoS ONE.

[31] Li K.K., Xia T., Ma F.M., Zhang R., Mao Y., Wang Y., Zhou L., Lau K.M., Ng H.K. (2015). miR-106b is overexpressed in medulloblastomas and interacts directly with PTEN. Neuropathol. Appl. Neurobiol..

[32] Shi J.A., Lu D.L., Huang X., Tan W. (2014). miR-219 inhibits the proliferation, migration and invasion of medulloblastoma cells by targeting CD164. Int. J. Mol. Med..

[33] Pan X., Wang Z., Wan B., Zheng Z. (2017). MicroRNA-206 inhibits the viability and migration of medulloblastoma cells by targeting LIM and SH3 protein 1. Exp. Ther. Med..

[34] Zhang Z.Y., Zhu B., Zhao X.W., Zhan Y.B., Bao J.J., Zhou J.Q., Zhang F.J., Yu B., Liu J., Wang Y.M. (2017). Regulation of UHRF1 by microRNA-378 modulates medulloblastoma cell proliferation and apoptosis. Oncol. Rep..

[35] Singh S.V., Dakhole A.N., Deogharkar A., Kazi S., Kshirsagar R., Goel A., Moiyadi A., Jalali R., Sridhar E., Gupta T. (2017). Restoration of miR-30a expression inhibits growth, tumorigenicity of medulloblastoma cells accompanied by autophagy inhibition. Biochem. Biophys. Res. Commun..

[36] Gao Y., Li P., Liu Z., Diao X., Song C. (2015). Expression levels of vascular endothelial cell growth factor and microRNA-210 are increased in medulloblastoma and metastatic medulloblastoma. Exp. Ther. Med..

[37] Pal R., Greene S. (2015). microRNA-10b Is Overexpressed and Critical for Cell Survival and Proliferation in Medulloblastoma. PLoS ONE.

[38] Kaid C., Silva P.B., Cortez B.A., Rodini C.O., Semedo-Kuriki P., Okamoto O.K. (2015). miR-367 promotes proliferation and stem-like traits in medulloblastoma cells. Cancer Sci..

[39] Wang C., Yun Z., Zhao T., Liu X., Ma X. (2015). MiR-495 is a Predictive Biomarker that Downregulates GFI1 Expression in Medulloblastoma. Cell Physiol. Biochem..

[40] Yogi K., Sridhar E., Goel N., Jalali R., Goel A., Moiyadi A., Thorat R., Panwalkar P., Khire A., Dasgupta A. (2015). MiR-148a, a microRNA upregulated in the WNT subgroup tumors, inhibits invasion and tumorigenic potential of medulloblastoma cells by targeting Neuropilin 1. Oncoscience.

[41] Panwalkar P., Moiyadi A., Goel A., Shetty P., Goel N., Sridhar E., Shirsat N. (2015). MiR-206, a Cerebellum Enriched miRNA Is Downregulated in All Medulloblastoma Subgroups and Its Overexpression Is Necessary for Growth Inhibition of Medulloblastoma Cells. J. Mol. Neurosci..

[42] Genovesi L.A., Carter K.W., Gottardo N.G., Giles K.M., Dallas P.B. (2011). Integrated analysis of miRNA and mRNA expression in childhood medulloblastoma compared with neural stem cells. PLoS ONE.

[43] Gokhale A., Kunder R., Goel A., Sarin R., Moiyadi A., Shenoy A., Mamidipally C., Noronha S., Kannan S., Shirsat N.V. (2010). Distinctive microRNA signature of medulloblastomas associated with the WNT signaling pathway. J. Cancer Res. Ther..

[44] Lv S.Q., Kim Y.H., Giulio F., Shalaby T., Nobusawa S., Yang H., Zhou Z., Grotzer M., Ohgaki H. (2012). Genetic alterations in microRNAs in medulloblastomas. Brain Pathol..

[45] de Biase D., Acquaviva G., Visani M., Sanza V., Argento C.M., De Leo A., Maloberti T., Pession A., Tallini G. (2020). Molecular Diagnostic of solid tumor using a Next Generation Sequencing custom-designed multi-gene panel. Diagnostics.

[46] Mi H., Muruganujan A., Thomas P.D. (2013). PANTHER in 2013: Modeling the evolution of gene function, and other gene attributes, in the context of phylogenetic trees. Nucleic Acids Res..

[47] Mi H., Huang X., Muruganujan A., Tang H., Mills C., Kang D., Thomas P.D. (2017). PANTHER version 11: Expanded annotation data from Gene Ontology and Reactome pathways, and data analysis tool enhancements. Nucleic Acids Res..

[48] Reimand J., Arak T., Adler P., Kolberg L., Reisberg S., Peterson H., Vilo J. (2016). g:Profiler-a web server for functional interpretation of gene lists (2016 update). Nucleic Acids Res..

[49] Chen E.Y., Tan C.M., Kou Y., Duan Q., Wang Z., Meirelles G.V., Clark N.R., Ma’ayan A. (2013). Enrichr: Interactive and collaborative HTML5 gene list enrichment analysis tool. BMC Bioinform..

[50] Kuleshov M.V., Jones M.R., Rouillard A.D., Fernandez N.F., Duan Q., Wang Z., Koplev S., Jenkins S.L., Jagodnik K.M., Lachmann A. (2016). Enrichr: A comprehensive gene set enrichment analysis web server 2016 update. Nucleic Acids Res..

[51] Poschl J., Stark S., Neumann P., Grobner S., Kawauchi D., Jones D.T., Northcott P.A., Lichter P., Pfister S.M., Kool M. (2014). Genomic and transcriptomic analyses match medulloblastoma mouse models to their human counterparts. Acta Neuropathol..

[52] Kool M., Jones D.T., Jager N., Northcott P.A., Pugh T.J., Hovestadt V., Piro R.M., Esparza L.A., Markant S.L., Remke M. (2014). Genome sequencing of SHH medulloblastoma predicts genotype-related response to smoothened inhibition. Cancer Cell..

[53] Northcott P.A., Korshunov A., Witt H., Hielscher T., Eberhart C.G., Mack S., Bouffet E., Clifford S.C., Hawkins C.E., French P. (2011). Medulloblastoma comprises four distinct molecular variants. J. Clin. Oncol..

[54] Livak K.J., Schmittgen T.D. (2001). Analysis of relative gene expression data using real-time quantitative PCR and the 2(-Delta Delta C(T)) Method. Methods.

[55] Szklarczyk D., Morris J.H., Cook H., Kuhn M., Wyder S., Simonovic M., Santos A., Doncheva N.T., Roth A., Bork P. (2017). The STRING database in 2017: Quality-controlled protein-protein association networks, made broadly accessible. Nucleic Acids Res..

[56] Shannon P., Markiel A., Ozier O., Baliga N.S., Wang J.T., Ramage D., Amin N., Schwikowski B., Ideker T. (2003). Cytoscape: A software environment for integrated models of biomolecular interaction networks. Genome Res..

[57] Kool M., Korshunov A., Remke M., Jones D.T., Schlanstein M., Northcott P.A., Cho Y.J., Koster J., Schouten-van Meeteren A., van Vuurden D. (2012). Molecular subgroups of medulloblastoma: An international meta-analysis of transcriptome, genetic aberrations, and clinical data of WNT, SHH, Group 3, and Group 4 medulloblastomas. Acta Neuropathol..

[58] Taylor M.D., Northcott P.A., Korshunov A., Remke M., Cho Y.J., Clifford S.C., Eberhart C.G., Parsons D.W., Rutkowski S., Gajjar A. (2012). Molecular subgroups of medulloblastoma: The current consensus. Acta Neuropathol..

[59] Northcott P.A., Hielscher T., Dubuc A., Mack S., Shih D., Remke M., Al-Halabi H., Albrecht S., Jabado N., Eberhart C.G. (2011). Pediatric and adult sonic hedgehog medulloblastomas are clinically and molecularly distinct. Acta Neuropathol..

[60] Ellison D.W., Kocak M., Dalton J., Megahed H., Lusher M.E., Ryan S.L., Zhao W., Nicholson S.L., Taylor R.E., Bailey S. (2011). Definition of disease-risk stratification groups in childhood medulloblastoma using combined clinical, pathologic, and molecular variables. J. Clin. Oncol..

[61] Christopher A.F., Kaur R.P., Kaur G., Kaur A., Gupta V., Bansal P. (2016). MicroRNA therapeutics: Discovering novel targets and developing specific therapy. Perspect Clin. Res..

[62] Chakraborty C., Sharma A.R., Sharma G., Doss C.G.P., Lee S.S. (2017). Therapeutic miRNA and siRNA: Moving from Bench to Clinic as Next Generation Medicine. Mol. Ther. Nucleic Acids.

[63] Visani M., de Biase D., Marucci G., Taccioli C., Baruzzi A., Pession A. (2013). Definition of miRNAs expression profile in glioblastoma samples: The relevance of non-neoplastic brain reference. PLoS ONE.

[64] McGlinn E., Yekta S., Mansfield J.H., Soutschek J., Bartel D.P., Tabin C.J. (2009). In ovo application of antagomiRs indicates a role for miR-196 in patterning the chick axial skeleton through Hox gene regulation. Proc. Natl. Acad. Sci. USA.

[65] Ma R., Yan W., Zhang G., Lv H., Liu Z., Fang F., Zhang W., Zhang J., Tao T., You Y. (2012). Upregulation of miR-196b confers a poor prognosis in glioblastoma patients via inducing a proliferative phenotype. PLoS ONE.

[66] Coskun E., von der Heide E.K., Schlee C., Kuhnl A., Gokbuget N., Hoelzer D., Hofmann W.K., Thiel E., Baldus C.D. (2011). The role of microRNA-196a and microRNA-196b as ERG regulators in acute myeloid leukemia and acute T-lymphoblastic leukemia. Leuk. Res..

[67] Lim J.Y., Yoon S.O., Seol S.Y., Hong S.W., Kim J.W., Choi S.H., Lee J.S., Cho J.Y. (2013). Overexpression of miR-196b and HOXA10 characterize a poor-prognosis gastric cancer subtype. World J. Gastroenterol..

[68] Kanno S., Nosho K., Ishigami K., Yamamoto I., Koide H., Kurihara H., Mitsuhashi K., Shitani M., Motoya M., Sasaki S. (2017). MicroRNA-196b is an independent prognostic biomarker in patients with pancreatic cancer. Carcinogenesis.

[69] Ge J., Chen Z., Li R., Lu T., Xiao G. (2014). Upregulation of microRNA-196a and microRNA-196b cooperatively correlate with aggressive progression and unfavorable prognosis in patients with colorectal cancer. Cancer Cell Int..

[70] Mo J.S., Alam K.J., Kang I.H., Park W.C., Seo G.S., Choi S.C., Kim H.S., Moon H.B., Yun K.J., Chae S.C. (2015). MicroRNA 196B regulates FAS-mediated apoptosis in colorectal cancer cells. Oncotarget.

[71] Ren D., Lin B., Zhang X., Peng Y., Ye Z., Ma Y., Liang Y., Cao L., Li X., Li R. (2017). Maintenance of cancer stemness by miR-196b-5p contributes to chemoresistance of colorectal cancer cells via activating STAT3 signaling pathway. Oncotarget.

[72] Lu Y.C., Chang J.T., Liao C.T., Kang C.J., Huang S.F., Chen I.H., Huang C.C., Huang Y.C., Chen W.H., Tsai C.Y. (2014). OncomiR-196 promotes an invasive phenotype in oral cancer through the NME4-JNK-TIMP1-MMP signaling pathway. Mol. Cancer.

[73] Cao D., Hu L., Lei D., Fang X., Zhang Z., Wang T., Lin M., Huang J., Yang H., Zhou X. (2015). MicroRNA-196b promotes cell proliferation and suppress cell differentiation in vitro. Biochem. Biophys. Res. Commun..

[74] Abe W., Nasu K., Nakada C., Kawano Y., Moriyama M., Narahara H. (2013). miR-196b targets c-myc and Bcl-2 expression, inhibits proliferation and induces apoptosis in endometriotic stromal cells. Hum. Reprod..

[75] Lv Z., Wei J., You W., Wang R., Shang J., Xiong Y., Yang H., Yang X., Fu Z. (2017). Disruption of the c-Myc/miR-200b-3p/PRDX2 regulatory loop enhances tumor metastasis and chemotherapeutic resistance in colorectal cancer. J. Transl. Med..

[76] Yao Y., Hu J., Shen Z., Yao R., Liu S., Li Y., Cong H., Wang X., Qiu W., Yue L. (2015). MiR-200b expression in breast cancer: A prognostic marker and act on cell proliferation and apoptosis by targeting Sp1. J. Cell Mol. Med..

[77] Ye F., Tang H., Liu Q., Xie X., Wu M., Liu X., Chen B. (2014). miR-200b as a prognostic factor in breast cancer targets multiple members of RAB family. J. Transl. Med..

[78] Hong H., Yu H., Yuan J., Guo C., Cao H., Li W., Xiao C. (2016). MicroRNA-200b Impacts Breast Cancer Cell Migration and Invasion by Regulating Ezrin-Radixin-Moesin. Med. Sci. Monit..

[79] Li D., Wang H., Song H., Xu H., Zhao B., Wu C., Hu J., Wu T., Xie D., Zhao J. (2017). The microRNAs miR-200b-3p and miR-429-5p target the LIMK1/CFL1 pathway to inhibit growth and motility of breast cancer cells. Oncotarget.

[80] Wu H., Wang G., Wang Z., An S., Ye P., Luo S. (2016). A negative feedback loop between miR-200b and the nuclear factor-kappaB pathway via IKBKB/IKK-beta in breast cancer cells. FEBS J..

[81] Rhodes L.V., Martin E.C., Segar H.C., Miller D.F., Buechlein A., Rusch D.B., Nephew K.P., Burow M.E., Collins-Burow B.M. (2015). Dual regulation by microRNA-200b-3p and microRNA-200b-5p in the inhibition of epithelial-to-mesenchymal transition in triple-negative breast cancer. Oncotarget.

[82] Kong X., Ding X., Li X., Gao S., Yang Q. (2015). 53BP1 suppresses epithelial-mesenchymal transition by downregulating ZEB1 through microRNA-200b/429 in breast cancer. Cancer Sci..

[83] Sun G., Cao Y., Wang P., Song H., Bie T., Li M., Huai (2018). miR-200b-3p in plasma is a potential diagnostic biomarker in oral squamous cell carcinoma. Biomarkers.

[84] Vosa U., Vooder T., Kolde R., Vilo J., Metspalu A., Annilo T. (2013). Meta-analysis of microRNA expression in lung cancer. Int. J. Cancer.

[85] Guan P., Yin Z., Li X., Wu W., Zhou B. (2012). Meta-analysis of human lung cancer microRNA expression profiling studies comparing cancer tissues with normal tissues. J. Exp. Clin. Cancer Res..

[86] Yu L., Todd N.W., Xing L., Xie Y., Zhang H., Liu Z., Fang H., Zhang J., Katz R.L., Jiang F. (2010). Early detection of lung adenocarcinoma in sputum by a panel of microRNA markers. Int. J. Cancer.

[87] de Bont J.M., Packer R.J., Michiels E.M., den Boer M.L., Pieters R. (2008). Biological background of pediatric medulloblastoma and ependymoma: A review from a translational research perspective. Neuro Oncol..

[88] Hartmann W., Koch A., Brune H., Waha A., Schuller U., Dani I., Denkhaus D., Langmann W., Bode U., Wiestler O.D. (2005). Insulin-like growth factor II is involved in the proliferation control of medulloblastoma and its cerebellar precursor cells. Am. J. Pathol..

[89] Borowska A., Jozwiak J. (2016). Medulloblastoma: Molecular pathways and histopathological classification. Arch. Med. Sci..

[90] Sandberg A.A., Stone J.F. (2008). The Genetics and Molecular Biology of Neural Tumors.

[91] Braicu C., Buse M., Busuioc C., Drula R., Gulei D., Raduly L., Rusu A., Irimie A., Atanasov A.G., Slaby O. (2019). A Comprehensive Review on MAPK: A Promising Therapeutic Target in Cancer. Cancers.

[92] MacDonald T.J., Brown K.M., LaFleur B., Peterson K., Lawlor C., Chen Y., Packer R.J., Cogen P., Stephan D.A. (2001). Expression profiling of medulloblastoma: PDGFRA and the RAS/MAPK pathway as therapeutic targets for metastatic disease. Nat. Genet..

[93] Park A.K., Lee J.Y., Cheong H., Ramaswamy V., Park S.H., Kool M., Phi J.H., Choi S.A., Cavalli F., Taylor M.D. (2019). Subgroup-specific prognostic signaling and metabolic pathways in pediatric medulloblastoma. BMC Cancer.

[94] Kieran M.W., Chisholm J., Casanova M., Brandes A.A., Aerts I., Bouffet E., Bailey S., Leary S., MacDonald T.J., Mechinaud F. (2017). Phase I study of oral sonidegib (LDE225) in pediatric brain and solid tumors and a phase II study in children and adults with relapsed medulloblastoma. Neuro Oncol..

[95] Meiss F., Andrlova H., Zeiser R. (2018). Vismodegib. Recent Results Cancer Res..

[96] Robinson G.W., Orr B.A., Wu G., Gururangan S., Lin T., Qaddoumi I., Packer R.J., Goldman S., Prados M.D., Desjardins A. (2015). Vismodegib Exerts Targeted Efficacy Against Recurrent Sonic Hedgehog-Subgroup Medulloblastoma: Results From Phase II Pediatric Brain Tumor Consortium Studies PBTC-025B and PBTC-032. J. Clin. Oncol..

[97] Lou E., Schomaker M., Wilson J.D., Ahrens M., Dolan M., Nelson A.C. (2016). Complete and sustained response of adult medulloblastoma to first-line sonic hedgehog inhibition with vismodegib. Cancer Biol. Ther..

[98] Li Y., Song Q., Day B.W. (2019). Phase I and phase II sonidegib and vismodegib clinical trials for the treatment of paediatric and adult MB patients: A systemic review and meta-analysis. Acta Neuropathol. Commun..

[99] Zhao X., Ponomaryov T., Ornell K.J., Zhou P., Dabral S.K., Pak E., Li W., Atwood S.X., Whitson R.J., Chang A.L. (2015). RAS/MAPK Activation Drives Resistance to Smo Inhibition, Metastasis, and Tumor Evolution in Shh Pathway-Dependent Tumors. Cancer Res..

[100] Gilbertson R.J., Langdon J.A., Hollander A., Hernan R., Hogg T.L., Gajjar A., Fuller C., Clifford S.C. (2006). Mutational analysis of PDGFR-RAS/MAPK pathway activation in childhood medulloblastoma. Eur. J. Cancer.

[101] Schuller U., Schober F., Kretzschmar H.A., Herms J. (2004). Bcl-2 expression inversely correlates with tumour cell differentiation in medulloblastoma. Neuropathol. Appl. Neurobiol..

[102] Mancarella C., Scotlandi K. (2019). IGF2BP3 From Physiology to Cancer: Novel Discoveries, Unsolved Issues, and Future Perspectives. Front. Cell Dev. Biol..

[103] Ramsay R.G., Gonda T.J. (2008). MYB function in normal and cancer cells. Nat. Rev. Cancer.

[104] Lassaletta A., Ramaswamy V. (2016). Medulloblastoma in adults: They’re not just big kids. Neuro Oncol..

[105] Lamm G.M., Christofori G. (1998). Impairment of survival factor function potentiates chemotherapy-induced apoptosis in tumor cells. Cancer Res..

[106] Call J.A., Naik M., Rodriguez F.J., Giannini C., Wu W., Buckner J.C., Parney I.F., Laack N.N. (2014). Long-term outcomes and role of chemotherapy in adults with newly diagnosed medulloblastoma. Am. J. Clin. Oncol..

[107] Brandes A.A., Ermani M., Amista P., Basso U., Vastola F., Gardiman M., Iuzzolino P., Turazzi S., Rotilio A., Volpin L. (2003). The treatment of adults with medulloblastoma: A prospective study. Int. J. Radiat. Oncol. Biol. Phys..

[108] Riffaud L., Saikali S., Leray E., Hamlat A., Haegelen C., Vauleon E., Lesimple T. (2009). Survival and prognostic factors in a series of adults with medulloblastomas. J. Neurosurg..

[109] Zhao F., Ohgaki H., Xu L., Giangaspero F., Li C., Li P., Yang Z., Wang B., Wang X., Wang Z. (2016). Molecular subgroups of adult medulloblastoma: A long-term single-institution study. Neuro Oncol..

[110] Ramaswamy V., Remke M., Bouffet E., Bailey S., Clifford S.C., Doz F., Kool M., Dufour C., Vassal G., Milde T. (2016). Risk stratification of childhood medulloblastoma in the molecular era: The current consensus. Acta Neuropathol..

[111] Shah M.Y., Ferrajoli A., Sood A.K., Lopez-Berestein G., Calin G.A. (2016). microRNA Therapeutics in Cancer—An Emerging Concept. EBio Med..

