# Correction: Visani et al. miR-196B-5P and miR-200B-3P Are Differentially Expressed in Medulloblastomas of Adults and Children. *Diagnostics* 2020, *10*, 265

**DOI:** 10.3390/diagnostics11091633

**Published:** 2021-09-07

**Authors:** Michela Visani, Gianluca Marucci, Dario de Biase, Felice Giangaspero, Francesca Romana Buttarelli, Alba Ariela Brandes, Enrico Franceschi, Giorgia Acquaviva, Alessia Ciarrocchi, Kerry Jane Rhoden, Giovanni Tallini, Annalisa Pession

**Affiliations:** 1 Department of Specialized, Diagnostic and Experimental Medicine, Anatomic Pathology-Molecular Diagnostic Unit AUSL-IRCCS of Bologna, University of Bologna School of Medicine, 40138 Bologna, Italy; giorgia.acquaviva3@unibo.it (G.A.); giovanni.tallini@unibo.it (G.T.); 2 Anatomic Pathology Unit, Ospedale Bellaria AUSL-IRCCS of Bologna, 40139 Bologna, Italy; Gianluca.Marucci@istituto-besta.it; 3 Department of Pharmacy and Biotechnology (FaBiT), Molecular Diagnostic Unit AUSL of Bologna, University of Bologna, 40138 Bologna, Italy; annalisa.pession@unibo.it; 4 Department of Radiological, Oncological and Anatomo-Pathological Sciences, Sapienza University School of Medicine, 00161 Rome, Italy; felice.giangaspero@uniroma1.it; 5 IRCCS Neuromed, 86077 Pozzilli (Isernia), Italy; 6 Department of Human Neurosciences, Sapienza University School of Medicine, 00161 Rome, Italy; francesca.buttarelli@uniroma1.it; 7 Department of Medical Oncology, Bellaria–Maggiore Hospitals AUSL-IRCCS of Bologna, 40139 Bologna, Italy; alba.brandes@yahoo.it (A.A.B.); enricofra@yahoo.it (E.F.); 8 Laboratory of Translational Research, Arcispedale Santa Maria Nuova AUSL-IRCCS of Reggio Emilia, 42122 Reggio Emilia, Italy; Alessia.Ciarrocchi@ausl.re.it; 9 Department of Medical and Surgical Sciences, Medical Genetics Unit, University of Bologna School of Medicine, 40138 Bologna, Italy; kerry.rhoden@unibo.it

The authors wish to make the following corrections to this paper [1]:

In the original article, there was a mistake in Figure 1 as published. The IHC of “WNT subgroup + GAB1” was incorrectly the same IHC as in “non-SHH/WNT subgroup+YAP1.” We probably made a mistake while assembling the picture. The corrected Figure 1 appears below. The authors apologize for any inconvenience caused and state that the scientific conclusions are unaffected. The original article has been updated.

## Figures and Tables

**Figure 1:**
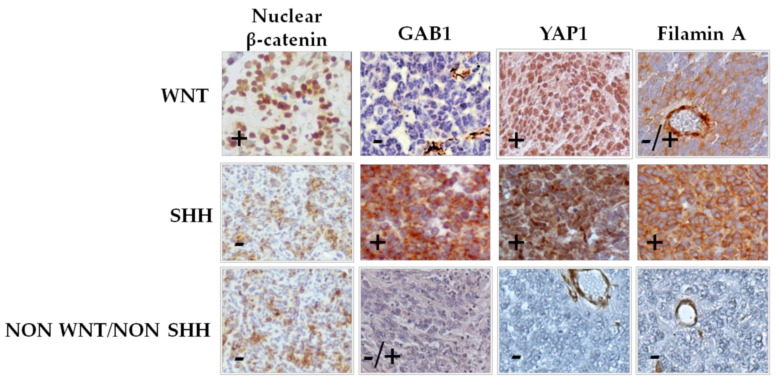
Classification of medulloblastoma by immunohistochemical staining. Cases were classified into WNT subgroup (nuclear β-catenin, positive; GAB1, negative; and YAP1, positive), SHH subgroup (nuclear β-catenin, negative; GAB1, positive; YAP1, positive; and filamin A, positive), and non-SHH/WNT subgroup (nuclear β-catenin, negative; YAP1, negative; and filamin A, negative). Representative pictures (40×) of each subgroup are shown.
