# Supplementary material for: miR-196B-5P and miR-200B-3P Are Differentially Expressed in Medulloblastomas of Adults and Children
Source: Diagnostics (Basel). 2020 Apr 29;10(5):265. doi: 10.3390/diagnostics10050265 (PMC7277606; doi:10.3390/diagnostics10050265)
Supplement: Supplementary file 1 [file diagnostics-10-00265-s001.zip › Supplementary Table S2.pdf]

Table S2. *CTNNB1* mutations founded in Adult and Childhood Medulloblastoma.

**Case**	***CTNNB1* Mutations**
AD-4	p.D32Y (c.94G>T)
AD-8	p.G34R (c.100G>A)
AD-10	p.D32E (c.96C>A)<br>p.V61F (c.181G>T)
AD-14	p.D32Y (c.94G>T)
CH-12	p.A13V (c.38A>G)
CH-15	p.S47N (c.140G>A)
CH-16	p.A20T (c.58G>A)
CH-20	p.D81N (c.241G>A)
